# Cognitive insights from tertiary sulci in prefrontal cortex

**DOI:** 10.1038/s41467-021-25162-w

**Published:** 2021-08-25

**Authors:** Willa I. Voorhies, Jacob A. Miller, Jewelia K. Yao, Silvia A. Bunge, Kevin S. Weiner

**Affiliations:** 1 Department of Psychology, University of California, Berkeley, CA USA; 2 Helen Wills Neuroscience Institute, University of California, Berkeley, CA USA; 3 Princeton Neuroscience Institute, Princeton University, Princeton, NJ USA

**Keywords:** Cognitive neuroscience, Problem solving

## Abstract

The lateral prefrontal cortex (LPFC) is disproportionately expanded in humans compared to non-human primates, although the relationship between LPFC brain structures and uniquely human cognitive skills is largely unknown. Here, we test the relationship between variability in LPFC tertiary sulcal morphology and reasoning scores in a cohort of children and adolescents. Using a data-driven approach in independent discovery and replication samples, we show that the depth of specific LPFC tertiary sulci is associated with individual differences in reasoning scores beyond age. To expedite discoveries in future neuroanatomical-behavioral studies, we share tertiary sulcal definitions with the field. These findings support a classic but largely untested theory linking the protracted development of tertiary sulci to late-developing cognitive processes.

## Abstract (web summary)

Tertiary sulci are shallow cortical folds that emerge late in gestation. Here the authors link prefrontal tertiary sulcal depth with reasoning scores in children and adolescents.

## Introduction

A fundamental question in cognitive neuroscience is how the structure of the brain supports complex cognition. While much progress has been made in answering this question, especially in animal models, human brains differ in both their micro- and macrostructural properties from widely used animals in neuroscience research such as mice, marmosets, and macaques^1^. These cross-species differences are especially pronounced in association cortices such as lateral prefrontal cortex (LPFC). LPFC is a late-developing cortical expanse that is enlarged in humans compared to non-human primates^2^ and is critical for cognitive control, executive function, reasoning, and goal-directed behavior^3–6^. Yet there is still much progress to be made in understanding how the development of evolutionarily new brain structures in the expanded human LPFC supports the development of complex, largely human, cognitive skills achieved by neural circuits within LPFC.

Of all the cognitive skills and anatomical features to focus on, we investigate the relationship between relational reasoning and macroanatomical structures in human cortex known as tertiary sulci. Sulci are commonly classified as primary, secondary, or tertiary based on their time of emergence in gestation^7–28^. Tertiary sulci are the last to emerge in utero, and subsequently are often the shallowest and smallest class of cortical folds^7–14,19,22–28^. They are largely overlooked due to methodological difficulties in their identification (which we expand on further below)^11,25,29^. Due to these difficulties, very little is known regarding the role of tertiary sulci in human cognition, despite the fact that many tertiary sulci are evolutionarily new structures. We refer to tertiary sulci as evolutionarily new because they are identifiable in humans and non-human hominoids (great apes), but not in other non-human primates^9,11,16–18,20,21,23,28,30^ (see Supplementary Information for further details regarding dimples and lack of tertiary sulci in non-human primates).

Here, we tested whether tertiary sulci in LPFC are behaviorally significant: that is, whether they relate to higher cognitive functioning. We focus on relational reasoning, which is the ability to extract common features across objects and conceptualize them in terms of their relation to each other^6,31^. Humans consistently outperform other species in tests of relational reasoning^6,32^, which relies on a distributed network involving LPFC that has expanded through primate evolution and that develops slowly over childhood and adolescence^6,33–38^. LPFC is considered critical to reasoning^6,37,39–43^ and developmental improvements in reasoning are associated with structural and functional connectivity between LPFC and lateral parietal cortex^32,37,39,44^.

As both reasoning and the LPFC exhibit protracted developmental trajectories in childhood, they serve as ideal targets to test a classic, yet largely unconsidered theory. Specifically, Sanides^9^ proposed that morphological changes in tertiary sulci would likely be associated with the slow development of higher-order thinking and cognitive skills^9,28^. Fitting these criteria, relational reasoning continues to develop throughout childhood, while tertiary sulci emerge late in gestation and continue to develop after birth for a still undetermined period of time^9,11,14,18,19,26,45,46^. A relationship between relational reasoning and tertiary sulcal morphology would build on previous findings relating the development of relational reasoning to changes in LPFC cortical thickness and structural connectivity^47,48^. Furthermore, relational reasoning supports complex problem solving and scaffolds the acquisition of additional cognitive skills in children^49,50^. Thus, exploring if or how tertiary sulci contribute to the development of this cognitive skill may not only provide insight into a classic theory, but also advance understanding of the anatomical features underlying variability in the development of a wide range of other cognitive skills.

While recent studies suggest a link between the morphology of tertiary sulci in association cortices and cognitive functions,^11,20,21,25,46,51^, no study to date (to our knowledge) has tested the role of tertiary LPFC sulci in cognitive development. This gap likely persists for three key reasons. First, previous studies examining individual differences in the development of reasoning and anatomical variability in human LPFC^39^ implemented analyses that were averaged across individuals on standard neuroanatomical templates, which obscure tertiary sulci in LPFC^25^ (Supplementary Figs. 1 and 2). Therefore, to precisely characterize the relationship between tertiary sulcal morphology in LPFC and reasoning performance, it is necessary to consider cortical anatomy at the level of the individual. Second, the shallowness of tertiary sulci makes them hard to reliably identify in post-mortem tissue—typically considered the gold standard for neuroanatomical analyses—because they are easily confused with shallow indentations produced by veins and arteries on the outer surface of the cerebrum^11,29^. Researchers interested in the function and structure of tertiary sulci have overcome this latter issue by (1) using T1 magnetic resonance images (MRI) and cortical surface reconstructions—either in vivo or post-mortem—to visualize tertiary sulci, and (2) manually tracing/defining tertiary sulci on either T1 MRI images or cortical surface reconstructions (“Methods”)^11,12,20,21,25,26,29,46,52–56^. Third, as detailed below, the patterning of LPFC tertiary sulci has classically remained contentious until recent studies^26,56,57^.

Indeed, earlier studies of LPFC sulcal patterning^22,24,27,58–62^ left tertiary LPFC sulci undefined or conflated with surrounding structures^21^ (“Methods”). For example, sulci consistent with the location of the modern definition of the posterior middle frontal sulcus (pmfs) were often considered the posterior end of the intermediate frontal sulcus (imfs)^60^. Functional and anatomical work by Petrides and colleagues^56,63^ has resolved these contentions by considering three components of the pmfs that are distinct from the imfs—a definition that additional recent work also supports^25^. The contention in classical definitions of tertiary sulci means that neuroanatomical atlases and neuroimaging software packages largely exclude tertiary sulci. In turn, tertiary sulci in LPFC have been excluded from most developmental cognitive neuroscience studies until the present study^11,20,21,25,46,51^. Nevertheless, there is increasing evidence that some tertiary sulci are functionally relevant in association cortices such as ventral temporal cortex^11^ (VTC), medial PFC^46,64^, and LPFC^25^ in adults, as well as behaviorally and clinically meaningful in medial PFC^16,20,55,65^. Irrespective of this mounting evidence that tertiary sulci are functionally and behaviorally relevant in association cortices within adults, it is largely unknown whether morphological features of tertiary sulci are linked to individual differences in behavior and cognition in a developmental cohort.

To address this gap in knowledge, we characterized LPFC tertiary sulci in a developmental sample. We studied a broad age range—children and adolescents between 6 and 18 years old—as we sought to leverage the neuroanatomical and cognitive variability intrinsically present in the sample in order to explore whether variability in tertiary sulcal morphology explains individual and developmental variability in relational reasoning. As sulcal depth is a characteristic feature of tertiary sulci, which are shallower than primary and secondary sulci^1,9,11,14,16,18,19,26,45^, we hypothesized a relationship between the depth of tertiary sulci and reasoning performance.

**Fig. 1:**
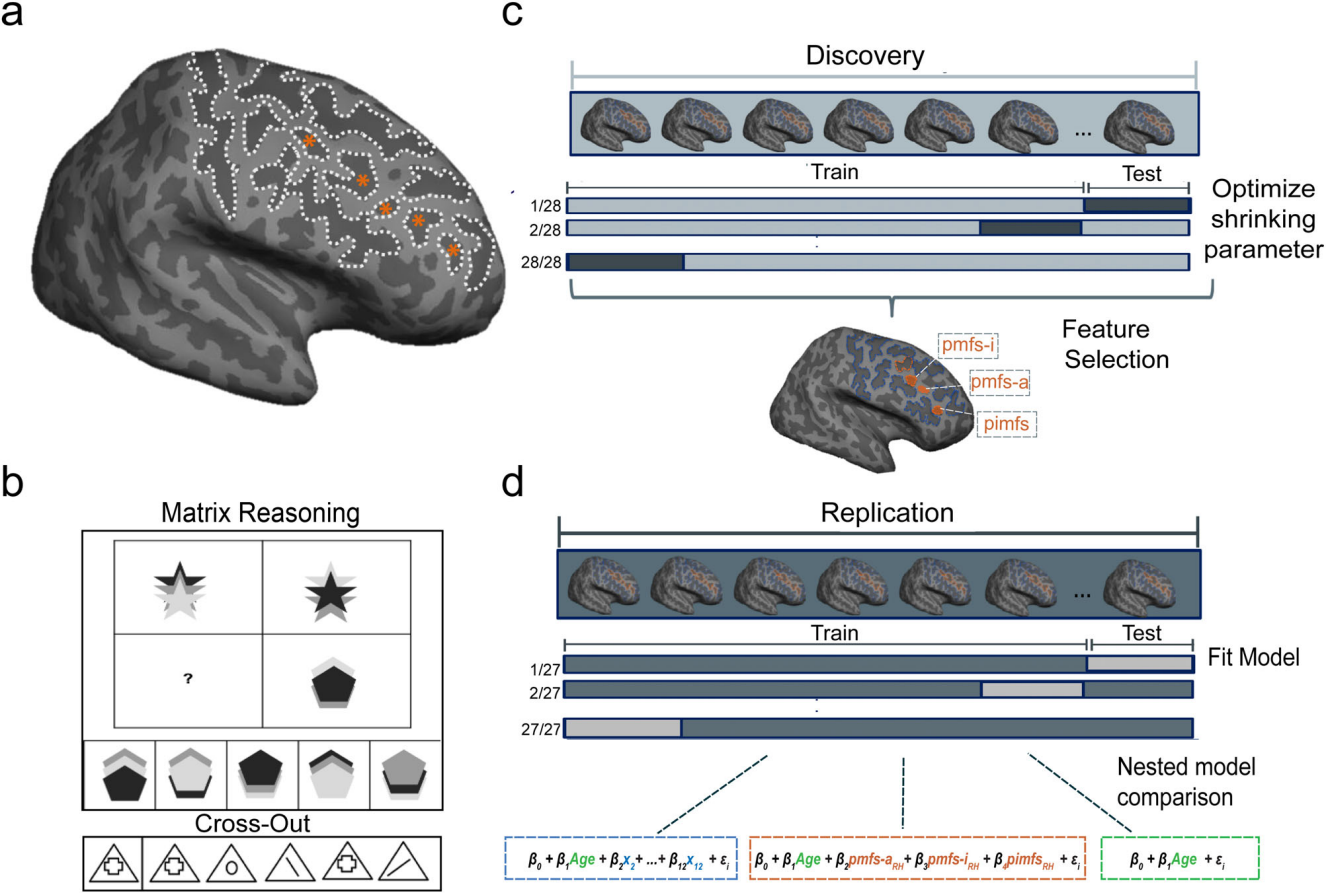
A data-driven analysis pipeline with Discovery and Replication samples that models the relationship between lateral prefrontal sulcal morphological features and reasoning performance. **a** An inflated cortical surface reconstruction of a right hemisphere from one example participant. Dotted white outlines show manually labeled sulci. Asterisks indicate the frequently omitted or misclassified tertiary sulci (Supplementary Fig. 1 for all participants). **b** Top: Example from the standardized test (modified from the original for display purposes) used to assess relational reasoning in this study (WISC-IV, Matrix reasoning task). In this task, participants are instructed to complete the matrix so that the relation between the two bottom shapes mirrors the relation between the two top shapes. In this example, option 4 completes the pattern. Bottom: Example from the processing speed task (WJ-R, Cross Out test), which serves as a behavioral control. In this task, participants are instructed to cross out all objects that match the object on the left as quickly as possible. **c** Feature selection—Discovery sample. A LASSO regression was performed in the Discovery sample to determine which sulci, if any, were associated with Matrix reasoning performance. The model parameters were fit iteratively using a leave-one-out cross-validation procedure (“Methods”). **d** Model evaluation—Replication sample. The sulci selected from the LASSO regression (orange; pmfs-i_RH_, pmfs-a_RH_, and pimfs_RH_) were included along with age in a model to predict task performance in the Replication sample. In order to assess the unique contribution of the selected sulci to task performance, this model (orange) was compared to two nested alternate models: (1) age alone (green) and (2) age in addition to all 12 labeled sulci (blue). All models were fit with a leave-one-out cross-validation procedure. pmfs-a: posterior middle frontal sulcus, anterior component; pmfs-i: posterior middle frontal sulcus, intermediate component; pmfs-p: posterior middle frontal sulcus, posterior component.

In this work, we developed a pipeline (Fig. 1) that combines the most recent anatomical definition of LPFC tertiary sulci^26^ with data-driven analyses to model sulcal morphological features and reasoning performance. We report three main findings. First, in our developmental sample, LPFC tertiary sulci can be reliably identified and are smaller, shallower, and more variable compared to primary LPFC sulci as in adults^25,26^. Second, there is a relationship between LPFC tertiary sulcal depth and reasoning performance across individuals such that a predictive model accurately captures the relationship between an individual’s LPFC tertiary sulcal depth and reasoning score above and beyond age in an independent sample. Third, this neuroanatomical-behavioral model does not generalize to other sulcal features or cognitive tasks. These findings quantitatively link LPFC tertiary sulcal morphology and reasoning performance, as well as provide cognitive insights from evolutionary new brain structures in LPFC.

## Results

### Tertiary sulci are consistently identifiable in the LPFC of 6–18 year-olds

**Fig. 2:**
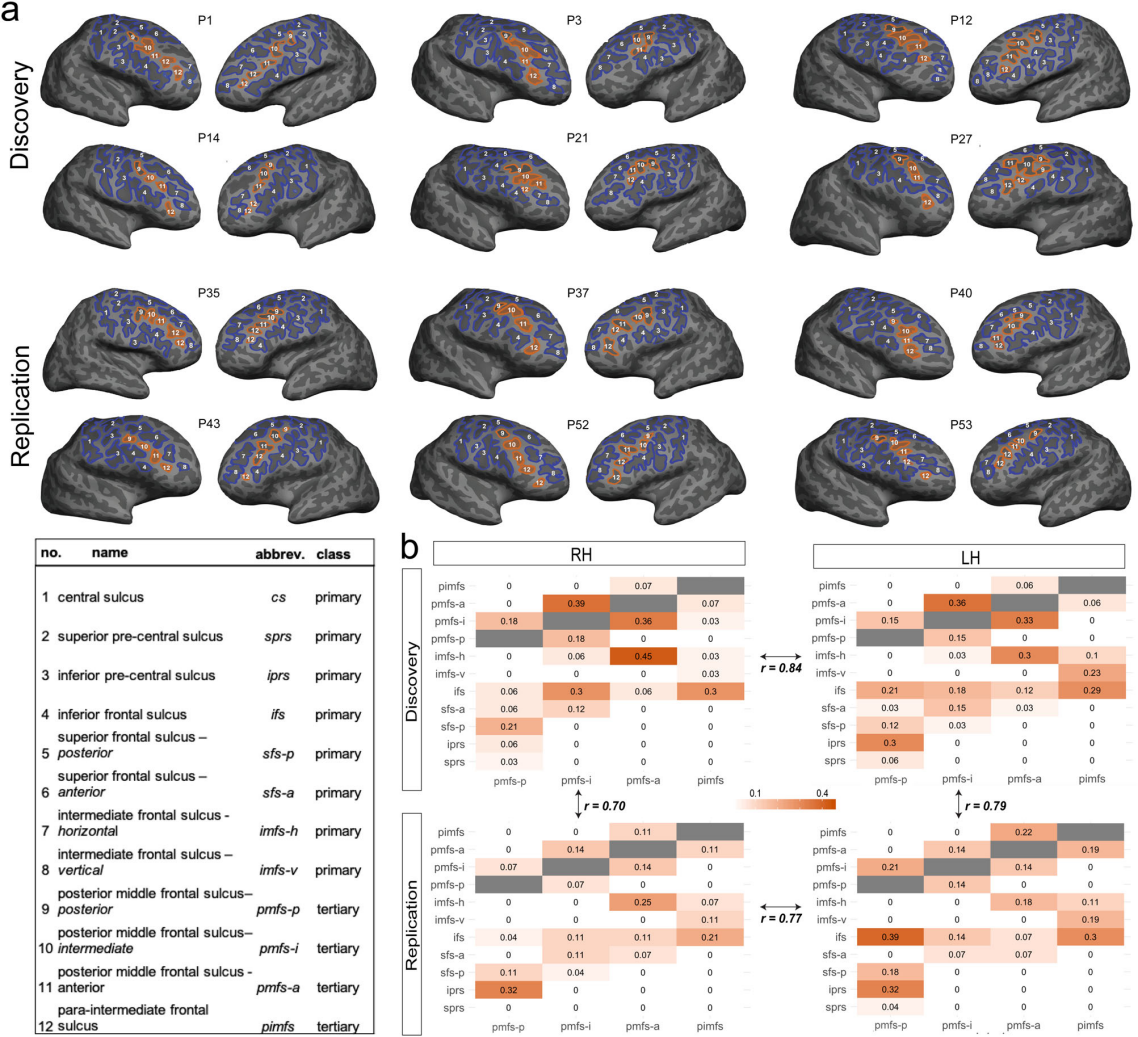
Lateral prefrontal tertiary sulci are identifiable and show comparable patterning across hemispheres and samples in children and adolescents. **a** Lateral prefrontal (LPFC) sulcal definitions on inflated cortical surface reconstructions from six example participants (P1, P3, etc.) in the Discovery sample (top) and the Replication sample (bottom). Sulci were identified based on the most recent neuroanatomical atlas to consider a comprehensive definition of tertiary sulci^26^. Primary sulci (1–8) are in blue, while tertiary sulci (9–12) are in orange. The three tertiary sulci (pmfs-i_RH_ (10), pmfs-a_RH_ (11), and pimfs_RH_ (12)) identified by our model-based approach with cross-validation (Fig. 4) are filled in. The distinction among primary, secondary, and tertiary sulci is based on classic and recent studies examining the timepoints when sulci emerge in gestation (“Methods” and Supplementary Information). Based on these studies, the sulci considered in the present work are either primary or tertiary (Supplementary Information). **b** Rates of intersection with surrounding sulci were quantified for each tertiary sulcus in the left (LH) and right (RH) hemispheres in order to identify common sulcal patterns. For each tertiary sulcus (pmfs-p (9), pmfs-i (10), pmfs-a (11), and pimfs (12)), we report the proportion of intersection (frequency of occurrence/total number of observations) with each LPFC sulcus (see color bar for reference; empty gray cells in the matrix reflect the fact that a sulcus cannot intersect with itself). Calculating the correlation between matrices shows that sulcal patterning is comparable (all *r*s > 0.70; all *p*s < 0.0001) between hemispheres and samples. Source data are provided as a Source data file.

Our sample consisted of 61 typically developing children and adolescents ages 6–18 years old. Participants were randomly assigned to Discovery (*N* = 33) and Replication (*N* = 28) samples with comparable age distributions (Discovery: mean(sd) = 12.0 (3.70); Replication: mean(sd) = 12.32 (3.53); *p* = 0.81). For each participant, we generated cortical surface reconstructions in FreeSurfer^66,67^ from high-resolution T1-weighted anatomical scans. As current automated methods do not define LPFC tertiary sulci and often include gyral components in sulcal definitions (Supplementary Fig. 2), all sulci were manually defined on the native cortical surface for each participant according to the most recent and comprehensive atlas of LPFC sulcal definitions^26^ (Fig. 2; Supplementary Fig. 1). LPFC sulci were classified as primary, secondary, or tertiary based on previous studies documenting the temporal emergence of sulci in gestation^10,19,21–25,27^. While the most modern sulcal parcellation was not included in these classic studies^10,19,22–25,27^, it is generally accepted that anterior middle frontal LPFC sulci emerge within the gestational window for primary sulci. Meanwhile, posterior LPFC middle frontal sulci emerge late in gestation^18,19,22^. Consequently, we designate posterior middle frontal sulci as tertiary, and all surrounding sulci as primary (see Fig. 2a for all classifications). We describe the criteria for classification and the correspondence between historical and contemporary sulcal definitions in more detail in the “Methods” and Supplementary Information.

We focused our analyses on the region commonly referred to as dorsal LPFC, which is bounded posteriorly by the central sulcus (cs), anteriorly by the horizontal (imfs-h), and ventral (imfs-v) components of the intermediate frontal sulcus, superiorly by the two components of the superior frontal sulcus (sfs-p and sfs-a), and inferiorly by the inferior frontal sulcus (ifs). Throughout the paper, we refer to this region as the LPFC (Fig. 2a). Studies in adults report as many as five tertiary sulci within these anatomical boundaries^26^: the three components of the posterior middle frontal sulcus (posterior: pmfs-p; intermediate: pmfs-i; anterior: pmfs-a) and the two components of the para-intermediate frontal sulcus (ventral: pimfs-v; dorsal: pimfs-d). We defined sulci on the inflated and pial cortical surfaces of each hemisphere for each participant (“Methods”). We emphasize that 1320 manual labels were created in total to examine the relationship between LPFC sulcal depth and reasoning performance (Supplementary Fig. 1 for sulcal definitions in all 122 hemispheres included in both samples). Sulcal definitions and all subsequent analyses are conducted separately for the Discovery and Replication samples, in order to assess the reliability and generalizability of our findings.

#### Discovery sample

All primary sulci—the central sulcus (cs), the superior (sprs) and inferior (iprs) portions of the precentral sulcus, as well as the sfs-p, sfs-a, ifs, imfs-h, and imfs-v—were identifiable in both hemispheres of each individual participant. We demonstrate that tertiary sulci in LPFC are consistently identifiable within the hemispheres of participants as young as 6 years old (Fig. 2a). The three components of the posterior middle frontal sulcus (pmfs-p; pmfs-i; pmfs-a) were identifiable in all participants in every hemisphere. However, the most anterior LPFC tertiary sulcus, the para-intermediate frontal sulcus (pimfs), was consistently variable across individuals (Supplementary Table 1). Specifically, while almost all participants had at least one identifiable component of the pimfs (right hemisphere: 30/33; left hemisphere: 31/33), we were only able to identify both dorsal and ventral pimfs components in 42.42% of all participants (right hemisphere: 12/33; left hemisphere: 16/33). We further quantify this variability in tertiary sulci by examining the prevalence of sulcal types, based on their rate of intersection with neighboring sulci (“Methods”; Fig. 2b). We find that sulcal patterning is very similar across hemispheres, with comparable rates of intersecting and independent sulci (*r* = 0.84, *p* < 0.0001).

#### Replication sample

Consistent with the Discovery sample, all primary sulci (numbered 1–8 in Fig. 2a) could be identified in both hemispheres of each individual participant. In terms of tertiary sulci, the pmfs-p, pmfs-i, and pmfs-a (numbered 9–11 in Fig. 2a) were also identifiable in each hemisphere of every individual. Once again, the pimfs was the most variable across individuals (Supplementary Fig. 1b; Supplementary Table 1). We were able to identify at least one pimfs component in almost every participant (right hemisphere: 28/28; left hemisphere: 27/28). Both the dorsal and ventral pimfs components were identifiable in 76.8% of hemispheres (right hemisphere: 19/28 participants; left hemisphere: 24/28; Supplementary Table 1). In each hemisphere, the rates and types of intersecting sulci were highly similar to those observed in the Discovery sample (right hemisphere: *r* = 0.70, left hemisphere: *r* = 0.79, all *p*s < 0.0001) and these were also consistent between hemispheres in this sample (*r* = 0.77, *p* < 0.0001; Fig. 2b).

In sum, we could identify LPFC tertiary sulci in both Discovery and Replication samples and found that the sulcal patterning was comparable—and highly correlated—between each sample. However, we could not identify both dorsal and ventral pimfs components in each hemisphere. Thus, our inclusion criterion for all subsequent analyses was to include participants who had at least one pimfs component in each hemisphere (Discovery: 28/33, Replication: 27/28), which assures that all repeated-measures statistics are balanced for effects of sulcus and hemisphere.

### LPFC tertiary sulci are shallower and more variable than primary sulci in children and adolescents

Classic anatomical studies report a high correspondence between sulcal classification and depth^9,14,18,19,45^, and recent in vivo studies in adults show that LPFC tertiary sulci are in fact significantly shallower and more variable than primary sulci in adults^25^. However, this correspondence has not been established for LPFC sulci in children and adolescents. Thus, we next sought to compare the depth and variability of LPFC tertiary and primary sulci in 6–18 year-olds. Sulcal depth was normalized to the maximum depth value within each individual hemisphere in order to account for differences in brain size across individuals and hemispheres (“Methods”). From these normalized measures, we conducted a two-way repeated-measures analysis of variance (rm-ANOVA) to statistically test for differences between sulcal type (primary, tertiary) and hemisphere (left, right) in both Discovery and Replication samples.

#### Discovery sample

**Fig. 3:**
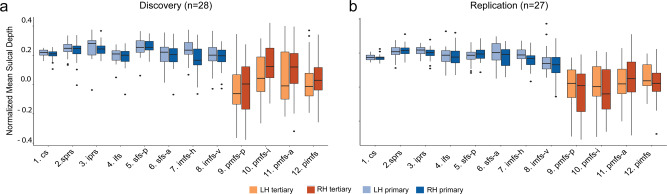
Lateral prefrontal tertiary sulci in 6–18 year-olds are more shallow and more variable than primary sulci. **a** Box-and-whisker plots showing Normalized sulcal depth for each of the 12 LPFC sulci in the Discovery sample for the left (LH) and right (RH) hemispheres. **b** Same as in (**a**), but for the Replication sample. Repeated-measures ANOVAs revealed that tertiary sulci (orange) were shallower and more variable than primary sulci (blue) in both samples (*p*s < 0.001). Horizontal lines represent median values and whisker lines represent the 1st and 3rd quartiles. Values falling outside of this range are represented as black dots. Source data are provided as a Source data file.

Consistent with findings in adults^25^, we observed a main effect of sulcal type (F(1,27) = 95.63, *p* *<* 10^−3^, *η*^2^G = 0.35) in which tertiary sulci were significantly more shallow than primary sulci (Mean(sd)Tertiary = 0.04(0.17); Mean(sd)Primary = 0.23(0.07)). We also observed an interaction between sulcal type and hemisphere (F(1,27) = 5.67, *p* < 0.02, *η*^2^G = 0.01) in which tertiary sulci were significantly deeper in the right hemisphere than in the left hemisphere (Mean(sd)_RH_ = 0.06(0.17); Mean(sd)_LH_ = 0.02(0.1)). In contrast, the depth of primary sulci did not differ between hemispheres (Mean(sd)_RH_ = 0.21(0.07); Mean(sd)_LH_ = 0.23(0.07)); Fig. 3a). To explore the morphological variability between sulcal types, we repeated the same analysis replacing mean sulcal depth with the standard deviation of sulcal depth. This analysis quantitatively supports that tertiary sulci are more variable than primary sulci (F(1,27) = 162.4, *p* *<* 10^−3^, *η*^2^G = 0.43), with no differences between hemispheres (*p* = 0.3).

#### Replication sample

We observed the same main effect of sulcal type in the Replication sample. Tertiary sulci were more shallow than primary sulci (F(1,26) = 136.5, *p* *<* 10^−3^, *η*^2^G = 0.46; Mean(sd)Tertiary = 0.02(0.16); Mean(sd)Primary = 0.23(0.07)). We did not observe an interaction with hemisphere in this sample (F(1,26) = 0.26, *p* = 0.62); Fig. 3b). Once again, an rm-ANOVA of the standard deviation of sulcal depth revealed that tertiary sulci were more variable than primary sulci across hemispheres (F(1,26) = 170.4, *p* *<* 10^−3^, *η*^2^G = 0.47).

In addition, while age was correlated with reasoning performance in both Discovery (*r* = 0.58, *p* < 10^−3^) and Replication samples (*r* = 0.73, *p* < 10^−3^), there was an inconsistent relationship between sulcal depth and age in either sample (Supplementary Fig. 3). Thus, we next implemented a two-pronged, model-based approach to test if including sulcal depth predicted reasoning score above and beyond age.

### A model-based approach with nested cross-validation reveals that including the depth of three LPFC tertiary sulci explains individual variability in reasoning above and beyond age alone

To examine the relationship between LPFC sulcal depth and reasoning scores, we implemented a data-driven pipeline with an emphasis on producing reliable and generalizable results. Based on current gold-standard recommendations^68^, we implemented a four-pronged analytic approach to assess and improve the generalizability of our results and each stage of analysis (“Methods”). First, we implemented a feature selection technique in the Discovery sample (Fig. 1c) to determine if the depths of any LPFC sulci are associated with reasoning performance (to remind the reader, we use depth in the model because this is the main morphological feature differentiating tertiary from primary sulci). To do so, we submitted sulcal depth values for all 12 LPFC sulci in the Discovery sample to a LASSO regression model, which provides an automated method for feature selection by shrinking model coefficients and removing sulci with very low coefficients from the model (Fig. 1c; “Methods”). This approach allowed us to determine, in a data-driven manner, which sulci are the strongest predictors of reasoning performance. In addition, this technique guards against overfitting and increases the likelihood that a model will generalize to other datasets, by providing a sparse solution that reduces coefficient values and decreases variance in the model without increasing bias^68,69^. Also, although we observe a gender imbalance in our samples, gender was not associated with sulcal depth (*p* = 0.27) or Matrix reasoning (*p* = 0.51); therefore, we do not consider gender further in our models.

**Fig. 4:**
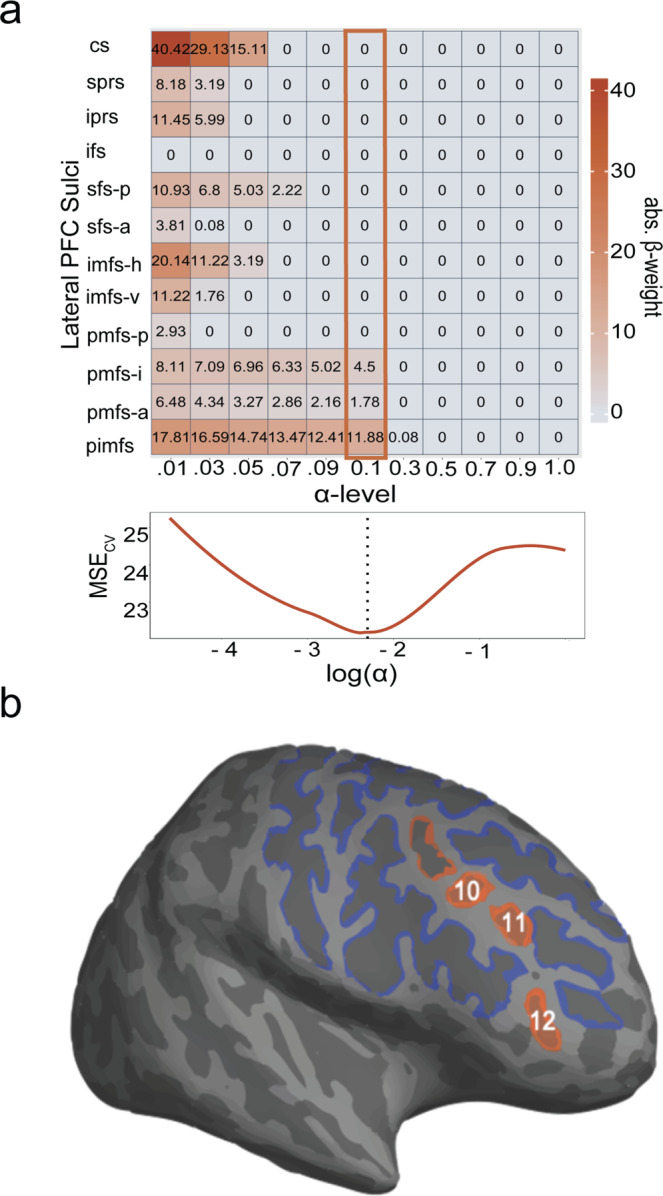
Data-driven model selection reveals that the depth of a subset of tertiary sulci is associated with reasoning. **a** Results from the LASSO regression predicting Matrix reasoning score from sulcal depth in the Discovery sample (*n* = 28 participants). Top: Beta-coefficients for each right hemisphere sulcus at a range of shrinking parameter (alpha) values. Highlighted box indicates coefficients at the chosen alpha-level. Bottom: Cross-validated mean-squared error (MSE_CV_) at each alpha-level. By convention^69^, we selected the *α* that minimized the MSE_CV_ (dotted line). **b** Inflated cortical surface from an example participant highlighting the three tertiary sulci (pmfs-i_RH_ (10), pmfs-a_RH_ (11), and pimfs_RH_ (12)) implicated in reasoning performance. Source data are provided as a Source Data file.

To determine the value of the shrinking parameter (*α*)^69^, we iteratively fit the model with a range of *α*-values using cross-validation. By convention^69^, we selected the *α* that minimized the cross-validated mean-squared error (MSECV; Fig. 4a). Although both tertiary and primary sulci were initially included as predictors, after implementing the LASSO regression, only three tertiary sulci (pmfs-i, pmfs-a, and pimfs) in the right hemisphere were found to be associated with reasoning performance (MSECV = 21.84, *α* = 0.1; *β*pmfs-i = 4.50, *β*pmfs-a = 1.78, *β*pimfs = 11.88; Fig. 4).

**Fig. 5:**
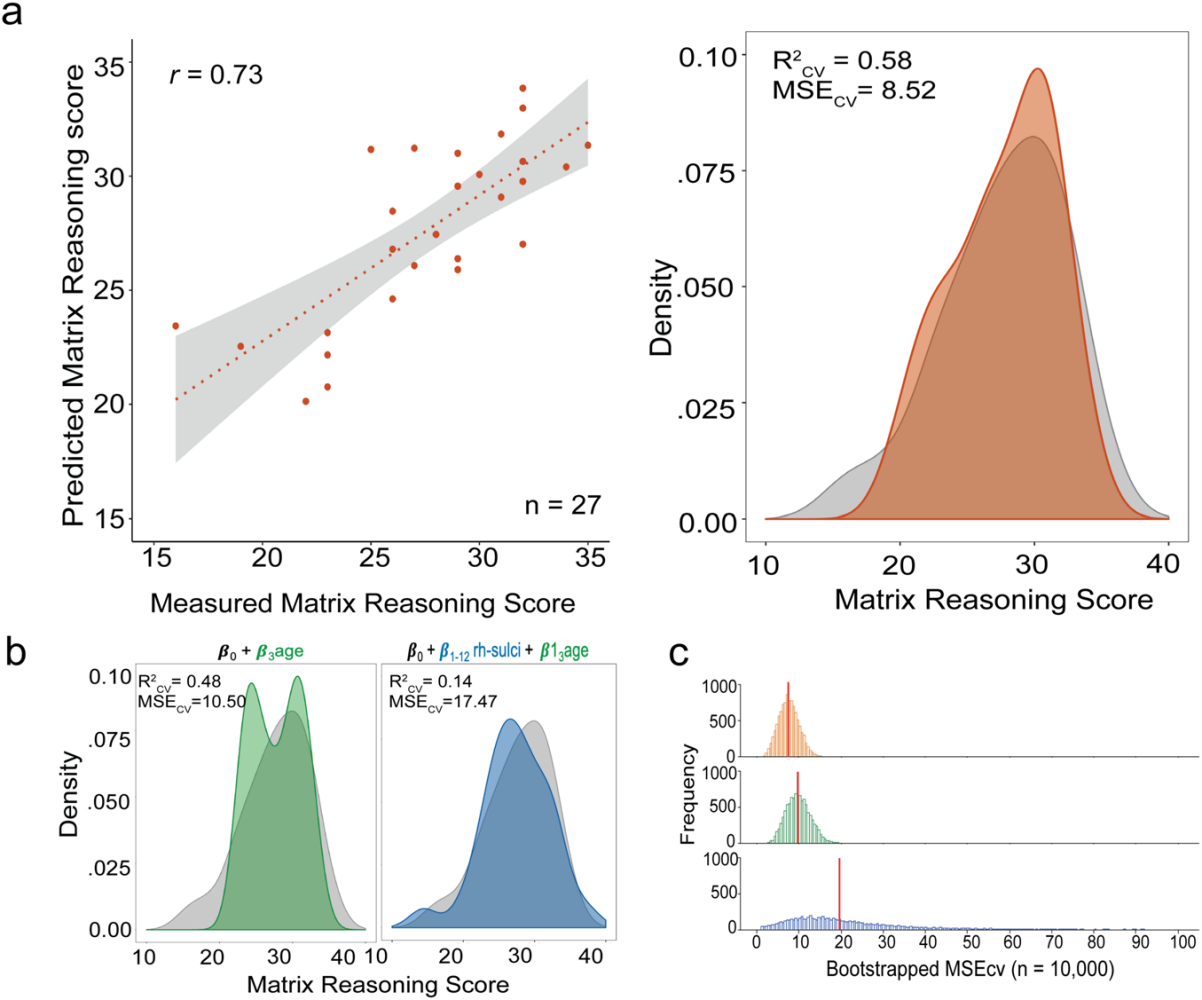
A model-based approach with nested cross-validation reveals that the depth of a subset of lateral prefrontal tertiary sulci explains individual variability in reasoning above and beyond age. **a** Left: Spearman’s correlation between measured and predicted Matrix reasoning scores in the Replication sample (*n* = 27 participants) for the best tertiary sulci + age model, which includes the depths of the two most relevant sulci (pmfs-i_RH_ + pimfs_RH_) from the Discovery sample, as well as age (Supplementary Fig. 5 for a model with all three tertiary sulci selected from the Discovery sample). Gray shading represents the 95% confidence interval for the linear model. Right: Density plot of model fit. The predicted scores from the chosen model (pmfs-i_RH_ + pimfs_RH_ + age) are shown in orange and overlaid on the distribution of measured Matrix reasoning scores (gray). **b** Distribution of predicted scores for the cross-validated nested model comparisons. Green: age only. Blue: all right hemisphere (RH) LPFC sulci + age. Each of the model fits is overlaid on the distribution of measured Matrix reasoning scores (gray). The pmfs-i_RH_ + pimfs_RH_ + age model (**a**) produced a better fit than both comparison models. Cross-validated mean-squared error (MSE_CV_) and model fit (*R*^2^_CV_) are reported for each of the three models. **c** Empirical MSE for each of the three models estimated with a bootstrapping procedure (*n*_iterations_ = 10,000) to address the potential for leave-one-out cross-validation to result in high variance and overfitting. The model including all LPFC RH sulci + age (blue) exhibited notably high variance in error estimation. The red vertical line indicates the estimated median MSE. Source data are provided as a Source data file.

To evaluate the generalizability of the sulcal-behavioral relationship identified in the Discovery sample, we constructed a linear model to predict reasoning score from sulcal depth and age in our Replication sample. The mean depths of the pmfs-i_RH_, pmfs-a_RH_, and pimfs_RH_, as well as age, were included as predictors in the model, as they were the only three sulci identified in the sulcal-behavioral model in the Discovery sample. As age was (as expected), highly associated with reasoning (Fig. 5a), including age in this model allowed us to compare performance of this tertiary sulci + age model to a model with age alone in order to determine the unique contribution of LPFC tertiary sulcal depth to reasoning performance above and beyond age. This model (and all subsequent models) were fit using a leave-one-out cross-validation (looCV) procedure. While looCV assesses the generalizability of the model within a sample and is appropriate for smaller sample sizes, it can result in models with high variance compared to other cross-validation techniques. To address this concern, we also estimated empirical MSE confidence intervals using a bootstrapping procedure (“Methods”). High variance in MSE across the bootstrapped iterations would suggest that the model is likely overfit to the original data.

We found that this model (pmfs-i_RH_ + pmfs-a_RH_ + pimfs_RH_ + age) was highly associated with reasoning score in the Replication sample (*R*^2^_CV_ = 0.52, MSE_CV_ = 9.66; Bootstrapped 95% CI_MSE_: 3.12–13.69, median_MSE_ = 8.14). In addition, we observed a high correspondence (Spearman’s rho = 0.70) between predicted and actual measured reasoning scores (Supplementary Fig. 5). Furthermore, if we consider just the two LPFC tertiary sulci that are the strongest predictors of reasoning performance as identified in the Discovery sample (pmfs-i_RH_: *β*_pmfs-i_ = 4.50; pimfs_RH_: *β*_pimfs_ = 11.88), the predictions of reasoning performance and model fits improved even further in the Replication sample (*R*^2^_CV_ = 0.58; MSE_CV_ = 8.52; Bootstrapped 95% CI_MSE_ = 3.21–12.37, median_MSE_ = 7.47; Spearman’s rho = 0.73; Fig. 5).

Once we had determined that the sulci relevant for reasoning in the Discovery sample were also associated with reasoning in the Replication sample, we used cross-validation to evaluate the fit of the replication model relative to two alternative models considering either (1) age alone or (2) sulcal depth from all right hemisphere LPFC sulci and age together in the Replication sample (Fig. 1d). This nested model comparison allowed us to determine the unique contribution of the depths of sulci identified by the model while still accounting for the effects of age and the depths of all LPFC sulci considered in the present study on reasoning performance. Removing the pmfs-i_RH_, pmfs-a_RH_, and pimfs_RH_ from the model decreased prediction accuracy and increased the MSE_CV_ (*R*^2^_CV_ = 0.48, MSE_CV_ = 10.50; Bootstrapped 95% CI_MSE_ = 4.69–15.67, median_MSE_ = 9.66), indicating that the depths of these right hemisphere tertiary sulci identified by our model-based approach explained a unique amount of variance in reasoning scores above and beyond age (Fig. 5b). In addition, considering age and the depths of all RH LPFC sulci also weakened the model prediction and increased MSE_CV_ (*R*^2^_CV_ = 0.14, MSE_CV_ = 17.47, Bootstrapped 95% CI_MSE_ = 2.79–306.25, median_MSE_ = 19.70). The bootstrapped CI_MSE_ showed that this model also suffered from very high variance (Fig. 5c). Taken together, our cross-validated, nested model comparison empirically supports that the depth of only a subset of LPFC tertiary sulci reliably explains unique variance in reasoning performance that is not accounted for by age or the depths of all LPFC sulci considered in the present study.

Finally, while our data demonstrate support for our hypothesis, we wondered whether our findings extended to other neuroanatomical features or related measures of cognitive development. We repeated our procedure with (1) a model in which we replaced sulcal depth with cortical thickness^70–73^ and (2) a model in which we replaced reasoning performance with performance on a behavioral measure that reflects a general cognitive ability: processing speed^74^. We used the Akaike Information Criterion (AIC) to quantitatively compare models. If the ∆AIC is >2, it suggests an interpretable difference between models. If the ∆AIC is >10, it suggests a strong difference between models, with the lower AIC value indicating the preferred model^75,76^ (“Methods”).

With respect to extension of these findings to another anatomical feature, this approach revealed that a model with cortical thickness and age was associated with reasoning (*R*^2^_CV_ = 0.33; MSE_CV_ = 13.54), but much less than the model with age alone (*R*^2^_CV_ = 0.48; MSE_CV_ = 10.50). The AIC for the thickness + age model (AIC_Thickness_ = 78.58) was much higher than the AIC for the tertiary sulci + age model (AIC_SulcalDepth_ = 63.85; ∆AIC_Thickness-Depth_ = 14.73). This indicates that sulcal depth is strongly preferred as a predictor over cortical thickness (Supplementary Fig. 6a).

To test whether sulcal depth was associated with another cognitive measure aside from reasoning, we used a test of processing speed (Cross Out^38^; Fig. 1b). Processing speed is a general cognitive ability that is correlated with—and theorized to support—reasoning^35,38,74,77^. As expected based on the prior literature, processing speed was correlated with reasoning performance in our sample (*rho* = 0.54, Supplementary Fig. 6c). Sulcal depth of the three critical LPFC tertiary sulci (pmfs-i_RH_, pmfs-a_RH_, and pimfs_RH_) and age was associated with processing speed (*R*^2^_CV_ = 0.45; MSE_CV_ = 20.53), but not much more than age alone (*R*^2^_CV_ = 0.42; MSE_CV_ = 21.82). The AIC for the processing speed + age model (AIC_CrossOut_ = 89.59) was much higher than the AIC for the tertiary sulci + age model (AIC_SulcalDepth_ = 63.85; ∆AIC_CrossOut - MatrixReasoning_ = 25.74), which indicates that reasoning is strongly preferred over processing speed (Supplementary Fig. 6b).

To further probe the relationship between these sulci and reasoning, we performed a follow-up analysis with a measure of phonological working memory (Digit Span Forwards) as another point of comparison. Like processing speed, working memory is a general cognitive ability that is correlated with—and theorized to support—reasoning^35,78^. As predicted based on the literature, our measures of reasoning and working memory were correlated (rho = 0.58; Supplementary Fig. 6c). However, the tertiary sulcal model (Model 1 detailed in the “Methods”) was not associated with phonological working memory (*R*^2^_CV_ = 0.10, MSE_CV_ = 2.75).

### Probability maps of LPFC sulci in children and adolescents

**Fig. 6:**
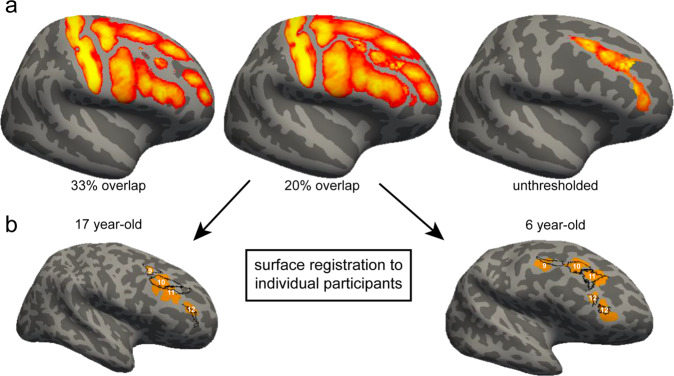
Probability maps of lateral prefrontal tertiary sulci. **a** Maximum probability maps were generated across all participants with at least one identifiable pimfs component in both hemispheres (*N* = 58). To generate the maps, each label was transformed from each individual to the common *fsaverage* space. For each vertex, we calculated the proportion of participants for whom that vertex is labeled as the given sulcus (the warmer the color, the higher the overlap in each image). In the case of multiple labels for one vertex, the sulcus with the highest overlap across participants was assigned to a given vertex. To reduce spatial overlap, these maps can be thresholded to only include vertices with a minimum percent overlap across participants (e.g., 33% (left) or 20% (middle) overlap). The maps of tertiary sulci within the middle frontal gyrus (MFG) in the present study (right, unthresholded) can be used in future studies to guide the definition of tertiary sulci within the MFG. **b** Maps can be projected to individual participants to guide the definition of tertiary sulci in lateral prefrontal cortex (LPFC). Here, the thresholded maps (20%) for the tertiary sulci are projected back to example (randomly chosen) hemispheres from a 17 year-old (left) and a 6 year-old (right). Outlines of the spatial probability maps (black) are overlaid on the manual sulcal definitions (orange) for visualization purposes. 9: pmfs-p; 10: pmfs-i; 11: pmfs-a; 12: pimfs. While there is not a perfect correspondence between the maps and the tertiary sulci, the maps can guide manual definitions performed by researchers interested in examining LPFC tertiary sulci in future studies. These maps can be applied to other samples and are available for download^109^.

As this is the first developmental dataset of tertiary sulci in LPFC (to our knowledge), we sought to generate spatial probability maps that can be shared with the field. The benefit of such maps is that they capture both the stable and variable features of LPFC sulci across participants. We calculated probability maps^25^ across all participants with at least one identifiable pimfs component in each hemisphere (*N* = 58). We provide examples of the unthresholded probability maps, which capture the spatial variability across participants, as well as maps thresholded at 20 and 33% overlap across participants (Fig. 6a). Thresholding captures the shared features across participants and can be applied to increase the interpretability and reduce spatial overlap between sulci^25^ (Methods). These probability maps can be projected to cortical surfaces in individual participants across ages (Fig. 6b) and can guide future research that aims to shed light on how LPFC tertiary sulcal morphology affects the functional organization in LPFC, as well as cognition.

## Discussion

Recent studies examining sulcal morphology in humans and other species continue to improve our understanding of the development and evolution of association cortices. They also provide anatomical insights into cognitive skills that set humans apart from other species^1,16,25,79^. A consistent finding from these previous studies is that developmentally and evolutionarily meaningful changes in sulcal morphology are not homogeneous within association cortices; instead, such changes are focal and related to different aspects of neuroanatomical and functional networks that are behaviorally meaningful^16,25,46,51,52,80–83^. After manually defining 1320 sulci in individual participants and implementing a data-driven approach with nested cross-validation in both Discovery and Replication samples, our results are consistent with and extend these previous findings by showing that the sulcal depth of particular LPFC tertiary sulci are linked to behavioral performance on a reasoning task in a developmental cohort, above and beyond age. In the sections below, we discuss (1) the identification of tertiary sulci in future studies, (2) potential underlying mechanisms that likely contribute to the relationship between tertiary sulcal depth and cognitive performance, (3) how the present findings provide a foundation for future studies attempting to link the morphology of brain structures to behavior and functional brain representations, and (4) how our model-based approach can be applied to study other association cortices across the lifespan.

While it may seem surprising that we were able to identify each pmfs component in every hemisphere, our findings are consistent with previous work showing that some tertiary sulci are identifiable in every hemisphere and others are not. For example, the mid-fusiform sulcus in ventral temporal cortex (VTC) is identifiable in every hemisphere in humans and non-human hominoids^11,12,30^, while the paracingulate sulcus is only identifiable in ~70% of hemispheres with a left hemisphere bias in medial prefrontal cortex in humans^20,46^ and only ~30% of the time in chimpanzees with no left hemisphere bias^16,20^. Consistent with recent findings in adult LPFC^21,25^, we could identify all three pmfs sulcal components in each hemisphere across participants in our developmental cohort. On the other hand, we could identify the pimfs components in a majority of participants, but not all. Thus, our findings are consistent with the previous literature regarding the definitions of tertiary sulci in different lobes. For instance, beyond LPFC and VTC, Lopez-Persem and colleagues^46^ found that some ventromedial PFC tertiary sulci are consistently identifiable, while other tertiary sulci are more variable. This is in line with our findings in LPFC—that the pmfs-p, pmfs-i, and pmfs-a are present in all participants, whereas the pimfs is variable in its presence and number of components.

An immediate question generated from our finding is Why might sulcal depth help to explain children and adolescents’ performance on a cognitive task, above and beyond age? We offer one potential explanation that integrates recent anatomical findings^25,84^ with a classic theory^9^ and propose a hypothesis linking sulcal depth to short-range anatomical connections, and in turn, to cortical networks and cognitive performance. Specifically, in the 1960s, Sanides^9,28^ proposed that morphological changes in tertiary sulci would likely be associated with the development of higher-order processing and cognitive skills. The logic of Sanides’ hypothesis extends from the fact that tertiary sulci emerge last in gestation and have a protracted development after birth, while complex cognitive skills such as reasoning ability also have a protracted development in childhood. Our findings support this classic hypothesis. However, while the LPFC is considered critical to reasoning^6,37,39–41^, reasoning performance cannot be localized to a single structure^6,37,39,85^ and thus, the mechanism behind this relationship still needs to be investigated.

As a starting point toward understanding the underlying mechanism, two recent empirical findings provide underlying anatomical mechanisms that could support this relationship between tertiary sulci and cognition. First, there is a relationship between human LPFC tertiary sulcal morphology and myelination^9,25,28^, which is critical for short- and long-range connectivity, as well as the efficiency of communicating neural signals among regions within cortical networks^86^. Second, anatomical work in non-human primates has shown that long-range white matter fiber tracts have a bias for terminating in gyri, while additional short-range white matter fibers commonly project from the deepest points (fundi) of sulci^84^, which we refer to as fundal fibers. These previous and present findings serve as the foundation for the following mechanistic hypothesis linking tertiary sulcal depth to anatomical connections and neural efficiency: deeper tertiary sulci likely reflect shorter fundal fibers, which in turn, reduce the length of short-range anatomical connections between cortical regions, and thus, increase neural efficiency. While speculative, this hypothesis is similar in logic to the tension-based theory of cortical folding^87^ and also feasible given the fact that short-range structural connectivity increases and sulci deepen during development^88,89^. This increase in neural efficiency could underlie variability in cognitive performance, which can be tested in future studies incorporating anatomical, functional, and behavioral measures, as well as computational modeling.

In addition to this mechanistic hypothesis, our present findings improve the spatial scale of previous studies attempting to link cortical morphology to behavior associated with LPFC. For example, previous studies identified an association between cognitive skills and cortical thickness of LPFC in its entirety^70–73^. While we find an association between reasoning and cortical thickness, when considering individual tertiary sulci, our analyses indicate that the depths of tertiary sulci and age together are much stronger predictors of reasoning than the cortical thickness of these sulci and age together. In fact, when including the cortical thickness of sulci in the model, performance was not better than age alone (Supplementary Fig. 6a). The combination of these findings across studies suggests that neuroanatomical-behavioral relationships can exist at multiple spatial scales in the same macroanatomical expanse such as LPFC: cortical thickness at the macroanatomical scale and tertiary sulcal depth at the meso-scale.

We also emphasize that, though our model-driven approach identified that the depth of a subset of LPFC tertiary sulci explained a significant amount of variance in individual reasoning scores above and beyond age, it is highly probable that these LPFC tertiary sulci are implicated in other tasks beyond reasoning—and, conversely, that other sulci are also implicated in reasoning. Although we did not observe a relationship between the depths of the identified sulci and two other cognitive measures, this should not be taken as evidence that these sulci show specificity to reasoning; rather, they indicate that these tertiary sulci are relevant for the task at hand.

We also clarify that the present approach of precise anatomical mapping of tertiary sulci does not imply that reasoning can be localized to a single sulcus, or even a single cortical region. In fact, our previous work, including previous studies on this dataset, has focused extensively on the distributed nature of reasoning, highlighting patterns of functional and structural connectivity between prefrontal and parietal regions that support this process^6,37,39^. In addition, focusing on tertiary sulci in PFC forms a foundation for understanding how these largely overlooked neuroanatomical structures contribute to typical brain function and cognition, especially at the network level^21^. Indeed, modern multi-modal neuroimaging research from two recent parallel lines of work show that meticulously labeling tertiary sulci within individuals uncovers structural-functional relationships within PFC at the network level^16,25^. For example, Miller and colleagues showed that each component of the pmfs participated in more than one network, indicating that these tertiary sulci also have flexible roles as members of different cognitive networks (e.g., ventral attention and cognitive control networks for the pmfs-a, for example). Thus, future studies exploring the relationship between sulcal morphology and behavioral performance in additional cognitive tasks at the level of individual participants will begin to generate a more comprehensive sulcal-behavioral map in LPFC with additional insights into cortical networks.

In addition to this sulcal-behavioral map in LPFC, two recent lines of work show feasibility for future studies attempting to link tertiary sulcal morphology to brain function, especially for functional activity related to reasoning: one related to tertiary sulci as a meso-scale link between microstructural and functional properties of LPFC and the other identifying functional representations related to reasoning. In terms of the former, a series of recent studies have shown that tertiary sulci are critical functional landmarks in different association cortices^12,13,46,65^ and variability in sulcal morphology in the medial prefrontal cortex has been associated with changes in cortical morphometry linked to individual differences in cognitive performance and clinical symptom presentation in patients with schizophrenia^20^. In addition, in LPFC, Miller and colleagues^25^ showed that the different *pmfs* components explored here were functionally distinct in adults with respect to resting-state connectivity profiles. In terms of the latter, numerous functional neuroimaging studies show that LPFC is central for reasoning performance^40,90^. More explicitly, several studies also indicate that the middle frontal gyrus, the gyrus in which the three sulci (pmfs-i, pmfs-a, and pimfs) identified by our model are located, plays an important role in cognitive processes that are integral for reasoning, such as maintaining representations and forming associations^3,4^. Thus, future investigations of functional connectivity, as well as functional representations, relative to tertiary sulci in future studies in children and adults will likely bring us closer to understanding the complex relationship between the development of LPFC anatomical organization, functional organization, and behavior.

While we limit our focus to the LPFC in the present study, both because of its relevance for reasoning, but also because of the immense manual labor involved in this type of study, the data-driven pipeline introduced here can be applied to any cortical expanse. For example, lateral parietal cortex is also critical for relational reasoning, is expanded in humans compared to non-human primates^2,91^, and also contains tertiary sulci^26^. In addition, structural connectivity between frontal and parietal regions increases across development^39,92,93^. Thus, future studies can explore how morphological features of tertiary sulci in (a) LPFC and lateral parietal cortex contribute to reasoning performance and (b) different association cortices contribute to performance on cognitive tasks, as well as functional representations in each cortical expanse. It will also be important to explore the relationship among tertiary sulci across cortical regions. For example, developmental studies are well suited to explore how the variability in sulcal morphology in one cortical region, such as LPFC, might affect morphology of tertiary sulci in other cortical regions, such as medial frontal or parietal regions. Our modeling approach can also be applied to data across the lifespan—either cross-sectionally or longitudinally. While it is known that tertiary sulci are shallow indentations in cortex that emerge last in gestation (relative to primary and secondary sulci), and have a protracted development after birth^1,9,11,14,16,18,19,26,45^, the history of LPFC sulcal definitions, especially within the MFG, has been contentious^7–14,19,21–28^. Thus, while we used these classic studies to guide the labeling of each sulcus, the distinctions among primary, secondary, and tertiary sulci should be confirmed by modern studies of cortical folding in gestation. Crucially, our findings are not dependent on this classification. Our data-driven, model-based approach identified that a subset of shallow sulci in LPFC explains the most variance in reasoning scores across participants above and beyond age in both Discovery and Replication samples. In addition, the developmental timeline of tertiary sulci relative to the development of functional representations and cognitive skills is unknown. Future studies implementing and improving our model-based approach can begin to fill in these gaps in the developmental timeline of tertiary sulci anatomically, behaviorally, and functionally.

Despite the many positive applications of our model-based approach and the many future studies that will likely build on the foundation of the present findings, there are also limitations. The main drawback of the precise approach in individual participants implemented here is that it relies on manual sulcal definitions, which are time-consuming and require anatomical expertise. This limits sample sizes and the expanse of cortex that can be feasibly explored in a given study. In addition, while there is “no one-size-fits-all sample size for neuroimaging studies”^94^ and we had a large *N* (>1000) in terms of sulci explored in the present study, new methods and tools will need to be developed to increase the number of participants in futures studies. Increasing the number of participants will improve the diversity of our sample and reduce imbalances in gender or other demographic features (“Methods”; Supplementary Table 2). Ongoing work is already underway to develop deep learning algorithms to accurately define tertiary sulci automatically in individual participants, and initial results are promising^53,95^. In the interim, our probabilistic sulcal maps can guide manual definitions performed by researchers interested in examining LPFC tertiary sulci in future studies (Fig. 6).

In summary, using a data-driven, model-based approach, we provide cognitive insights from evolutionarily new brain structures in human LPFC. After manually defining 1320 LPFC sulci, our approach revealed that the depths of a subset of tertiary sulci reliably explained unique variance in reasoning scores above and beyond age. Methodologically, our study opens the door for future studies examining tertiary sulci in other association cortices, as well as improves the spatial scale of understanding for future studies interested in linking cortical morphology to behavior. Theoretically, the present results support a largely unconsidered anatomical theory proposed over 55 years ago^9^. Mechanistically, we outline a hypothesis linking tertiary sulcal depth to short-range white matter fibers, neural efficiency, and cognitive performance. Together, the methodological, theoretical, and mechanistic insights regarding whether, or how, tertiary sulci contribute to the development of higher-level cognition in the present study serve as a foundation for future studies examining the relationship between the development of cognitive skills and the morphology of tertiary sulci in association cortices more broadly.

## Methods

### Participants

The present study consisted of Discovery (*N* = 33; 16 males and 17 females) and Replication (*N* = 28; 20 males and 8 females) samples. For the Discovery sample, 33 typically developing individuals between the ages of 6–18 were randomly selected from the Neurodevelopment of Reasoning Ability (NORA) dataset^37,38,44^. Demographic and socioeconomic data are summarized in Supplementary Table 2. Following the definition of sulci in this sample, we selected an additional 28 age-matched participants for the Replication sample. No features other than age were considered in the selection of the Replication sample. The terms male and female are used to denote parent-reported gender identity. All participants were screened for neurological impairments, psychiatric illness, history of learning disability, and developmental delay. All participants and their parents gave their informed assent and/or consent to participate in the study, which was approved by the Committee for the Protection of Human Participants at the University of California, Berkeley.

### Data acquisition

#### Imaging data

Brain imaging data were collected on a Siemens 3T Trio system at the University of California Berkeley Brain Imaging Center. High-resolution T1-weighted MPRAGE anatomical scans (TR = 2300 ms, TE = 2.98 ms, 1 × 1 × 1 mm voxels) were acquired for cortical morphometric analyses.

#### Behavioral data

Behavioral metrics are only reported for the participants included in the morphology-behavior analyses (Discovery: *n* = 28, Replication: *n* = 27). Reasoning performance was measured as a total raw score from the WISC-IV Matrix reasoning task^96^ (Fig. 1b; Discovery: mean(sd) = 24.28 (4.86); Replication: mean(sd) = 27.64 (4.52)). Matrix reasoning is an untimed subtest of the WISC-IV in which participants are shown colored matrices with one missing quadrant. The participant is asked to “complete” the matrix by selecting the appropriate quadrant from an array of options (Fig. 1b). Matrix reasoning score was selected as it is a widely used measure of non-verbal reasoning^37,38^ and it was the most consistently available reasoning measure for the participants in this study. Matrix reasoning has previously been examined in relation to white matter and functional connectivity in a large dataset that included these participants^37^ and a previous factor analysis in this dataset showed that the Matrix reasoning score loaded strongly onto a reasoning factor that included three other standard reasoning assessments^38^.

Processing speed was computed from raw scores on the Cross Out task from the Woodcock-Johnson Psychoeducational Battery-Revised^97^ (WJ-R; Fig. 1b). In this task, the participant is presented with a geometric figure on the left followed by 19 similar figures. The participant places a line through each figure that is identical to the figure on the left of the row (Fig. 1b). Performance is indexed by the number of rows (out of 30 total rows) completed in 3 minutes (Replication: Mean(sd) = 22.19 (6.26)). Cross Out scores are frequently used to estimate processing speed in developmental populations^98,99^.

As an additional measure, working memory (WM) was assessed from raw Digit Span Forward scores (Replication: Mean(sd) = 9.03(1.77)). Digit Span Forward scores measure WM maintenance and attention. For each forward trial, participants were presented with a string of numbers by the experimenter and were asked to immediately repeat the numbers in the same order. The task consisted of eight questions with two trials per level (16 total trials). Each question (set of two trials) consisted of a longer string of numbers than the question before. Both processing speed and working memory were selected as they are considered related, but separable, measures from reasoning. We report the Spearman correlation coefficient (rho) among each of the three behavioral measures in Supplementary Fig. 6.

### Morphological analyses

#### Cortical surface reconstruction

All T1-weighted images were visually inspected for scanner artifacts. FreeSurfer’s automated segmentation tools^66,67^ (FreeSurfer 6.0.0) were used to generate cortical surface reconstructions. Each anatomical T1-weighted image was segmented to separate gray from white matter, and the resulting boundary was used to reconstruct the cortical surface for each participant^66,100^. Each reconstruction was visually inspected for segmentation errors, and these were manually corrected when necessary. Tertiary sulci are easier to identify on T1 images and cortical surface reconstructions compared to post-mortem tissue (see the “Introduction”) for two main reasons. First, T1 MRI protocols are not ideal for imaging vasculature; thus, the vessels that typically obscure the tertiary sulcal patterning in post-mortem brains are not imaged on standard resolution T1 MRI scans. Indeed, indentations produced by these smaller vessels that obscure the tertiary sulcal patterning are visible in freely available datasets acquired at high field (7T) and micron resolution (100–250 μm)^101,102^. Thus, the present resolution of our T1s (1 mm isotropic) is sufficient to detect the shallow indentations of tertiary sulci, but is not confounded by smaller indentations produced by vasculature. Second, cortical surface reconstructions are made from the boundary between gray and white matter; unlike the outer surface, this inner surface is not obstructed by veins and arteries^11,29^.

#### Manual labeling of LPFC sulci

Sulci were manually defined separately in the Discovery and Replication samples according to the most recent atlas proposed by Petrides^26^. This atlas offers a comprehensive schematization of sulcal patterns in the cerebral cortex. The LPFC definitions have recently been validated in adults^25^, but to our knowledge, these sulci have never been defined in a developmental sample. 12 LPFC sulci were manually defined within each individual hemisphere in tksurfer^25^ (Fig. 2; Supplementary Fig. 1 for all manually defined sulci in 122 hemispheres). Sulcal depth values are a feature of FreeSurfer’s scale, which can be explored further on their website (https://surfer.nmr.mgh.harvard.edu). Briefly, depth values are calculated based on how far removed a vertex is from what is referred to as a “mid-surface,” which is determined computationally so that the mean of the displacements around this “mid-surface” is zero. Thus, generally, gyri have negative values, while sulci have positive values. Given the shallowness and variability in the depth of LPFC tertiary sulci, some mean depth values extend below zero. We emphasize that this just reflects the metric implemented in FreeSurfer. For example, max depth values are above zero for all sulci (Supplementary Fig. 4b). Manual lines were drawn on the inflated cortical surface to define sulci based on the proposal by Petrides^26^ as well as guided by the pial and smoothwm surfaces of each individual^25^. In some cases, the precise start or end point of a sulcus can be difficult to determine on one surface^53^. Thus, using the inflated, pial, and smoothwm surfaces of each individual to inform our labeling allowed us to form a consensus across surfaces and clearly determine each sulcal boundary. Our cortical expanse of interest was bounded by the following sulci: (1) the anterior and posterior components of the superior frontal sulcus (sfs) served as the superior boundary, (2) the inferior frontal sulcus (ifs) served as the inferior boundary, (3) the central sulcus served as the posterior boundary, and (4) the vertical and horizontal components of the intermediate frontomarginal sulcus (imfs) served as the anterior boundary. We also considered the following tertiary sulci: anterior (pmfs-a), intermediate (pmfs-i), and posterior (pmfs-p) components of the posterior middle frontal sulcus (pmfs), and the para-intermediate frontal sulcus (pimfs)^25,26^. Please refer to Fig. 2a for the location of each of these sulci on example hemispheres and Supplementary Fig. 1 for the location of all 1320 sulci in all 122 hemispheres. For each hemisphere, the location of each sulcus was confirmed by two trained independent raters (W.V. and J.Y.) and finalized by a neuroanatomist (K.S.W.). The surface vertices for each sulcus were then manually selected using tools in tksurfer and saved as surface labels for vertex-level analyses of morphological statistics. All anatomical labels for a given hemisphere were fully defined before any morphological or behavioral analyses were performed.

While we could not identify the dorsal and ventral components of the pimfs in every hemisphere (see the “Results” section; Supplementary Table 1), we could identify at least one component of the pimfs in each hemisphere in nearly all participants in the Discovery (28/33) and Replication (27/28) samples. Thus, our inclusion criterion for all subsequent analyses was to include participants who had at least one pimfs component in each hemisphere, which assures that all repeated-measures statistics are balanced for effects of sulcus and hemisphere. For those participants who had identifiable dorsal and ventral pimfs components, we merged the components into one label, using the FreeSurfer function mris_mergelabels and all findings are reported for the merged label^66^.

#### Characterization of tertiary sulcal patterning

For each tertiary sulcus, we characterized sulcal patterns, or types, based on intersections with surrounding sulci. We report the number of intersections for a given sulcus with every other sulcal pair (except the central sulcus as no tertiary sulcus intersected with the central sulcus), relative to the total frequency of occurrence of that sulcus in the hemisphere (Fig. 2b). We report correlations between left and right hemispheres in each sample, as well as the correlation between samples.

#### Sulcal probability maps

Sulcal probability maps were calculated to describe the vertices with the highest and lowest correspondence across participants^25^. These maps were generated across all participants with at least one pimfs component in each hemisphere. To generate the maps, each label was transformed from each individual to the common *fsaverage* space. We chose to use the standard *fsaverage* template to increase accessibility for future studies. Then, for each vertex, we calculated the proportion of participants for whom that vertex is labeled as the given sulcus. In the case of multiple labels, labels were assigned to each vertex with a “winner-take-all” approach. That is, the sulcus with the highest overlap across participants was assigned to a given vertex. Consistent with Miller et al.^25^, in addition to providing unthresholded maps, we also constrained these maps to be maximum probability maps (MPMs), which helps avoid overlapping sulci and can increase interpretability (Fig. 6a)^25^. We provide thresholded maps at 33 and 20% spatial overlap for each label. This allows the user to assess both the spatial variability between participants as well as the stable features shared across participants. Finally, since this is the first developmental dataset of tertiary sulci in the frontal lobe, we make these maps publicly available for download at the following link: https://github.com/cnl-berkeley/stable_projects/tree/main/CognitiveInsights_SulcalMorphology.

#### Characterization of sulcal morphology

As the most salient morphological feature of tertiary sulci is their shallowness compared to primary and secondary sulci^9,11,14,18,19,26,29,46^, we focused morphological analyses on measures of sulcal depth. Raw depth metrics (standard FreeSurfer units) were computed in native space from the .sulc file generated in FreeSurfer 6.0.0^66^. We normalized sulcal depth to the maximum depth value within each individual hemisphere in order to account for differences in brain size across individuals and hemispheres. All depth analyses were conducted for normalized mean sulcal depth. As cortical thickness is a commonly used metric in developmental studies, we also considered the mean cortical thickness (mm) for each sulcus. Mean cortical thickness for each sulcal label was extracted using the mris_anatomical_stats function that is included in FreeSurfer^67^.

#### Distinction among primary, secondary, and tertiary sulci

Tertiary sulci are defined as the last sulci to emerge in gestation after the larger and deeper primary and secondary sulci^7–14,19,21–27^ (Fig. 2a). Specifically, previous studies specify that (a) primary sulci emerge prior to 32 weeks in gestation, (b) secondary sulci emerge between 32–36 weeks in gestation, and (c) tertiary sulci emerge during and after 36 weeks^103^ (see Supplementary Information for direct quotations describing the developmental timeline for primary, secondary, and tertiary sulci). Previous research identifies the cs, prs, sfs, and ifs as primary sulci. As such, we apply these definitions to the subcomponents of the sfs (sfs-a and sfs-p) and prs (sprs and iprs) considered here.

Apart from these sulci, the question of whether or not other LPFC sulci should be considered secondary or tertiary is still unresolved. For example, the imfs-v and imfs-h are contemporary labels for classic definitions of sulci commonly labeled as either the frontomarginal and/or middle frontal sulci^21,25,26^. When considering classic papers and atlases^10,22,24,27^, both the imfs-h and imfs-v appear to be prevalent prior to 32 weeks, which would define them as primary sulci. Yet, additional studies define sulci in this cortical expanse as secondary^15^. For the present study, we consider the imfs-h and imfs-v as primary sulci, but it is possible that future studies will establish them as secondary sulci. Critically, our data-driven approach—and in turn, our findings—are agnostic to these distinctions. That is, the model-based approach adopted here quantitatively determines which sulci are most strongly associated with reasoning scores, regardless of their classification. Finally, while historical analyses have not considered modern definitions of pmfs and pimfs sulcal components, more recent studies from Petrides and colleagues do. Moreover, these studies show a correspondence between LPFC tertiary sulcal definitions and brain activation profiles. For example, Amiez and Petrides^56^ showed that the pimfs and pmfs-a co-localize with clusters of fMRI activation with distinct functional profiles on a serial order memory task. Consistent with this work, Champod and Petrides^104^ also show a direct relationship between activation profiles and tertiary sulcal patterning within LPFC. Considering these data, we refer to pmfs and pimfs sulcal components as tertiary sulci—for two main reasons. First, from our historical analyses, sulci within the middle frontal gyrus emerge during late stages in gestation, consistent with definitions of tertiary sulci^21^. Second, from our previous analyses in adults^25^, pmfs sulcal components are small and shallow relative to other primary and secondary LPFC sulci, which is consistent with morphological features of tertiary sulci. Taken together, our distinction among primary and tertiary sulci is based on classic and modern data. Future studies with larger sample sizes using non-invasive fetal imaging will re-visit the timestamps for the documented sulci, as well as provide new timestamps for those sulci that were not included in these classic studies. For example, based on these classic definitions of sulcal types, the present study did not include any secondary sulci in LPFC. Nevertheless, we also highlight that our data-driven approach is blind to these definitions and identifies sulci that are small in surface area and shallow in depth, which is consistent with the definition of tertiary sulci.

#### Comparison between tertiary and primary sulci

We compared sulcal depth of tertiary and primary sulci with a two-way (hemisphere, sulcal type) repeated-measures analysis of variance (rm-ANOVA; Fig. 3). To assess the variability in depth between hemispheres and groups, we conducted the same rm-ANOVA, but replaced mean sulcal depth with the standard deviation. We conducted the same repeated-measures analyses with cortical thickness between tertiary and primary sulci in both samples (Supplementary Fig. 4; see Supplementary Information). All ANOVAs were computed in R with the *aov* function, imported in python via rpy2. Effect sizes are reported with the generalized eta-squared (*η*^2^) metric.

### Assessing the relationship between sulcal depth and reasoning performance

#### Four-pronged analytic approach

Based on current recommendations^68^, we implement a four-pronged approach to assess and improve the generalizability of our findings at each stage of analysis.

1. Regularization: In the Discovery sample, we use L1 regularization (LASSO regression) as part of our model-selection approach. Not only does this provide a data-driven method for model selection, but regularization techniques are recommended to improve the generalizability of a model^68,69^. Unlike many techniques that only assess generalizability, L1 regularization increases the generalizability of a model by providing a sparse solution that reduces coefficient values and decreases variance in the model without increasing bias. This technique guards against overfitting and increases the likelihood that a model will generalize to other datasets.
2. Cross-validation: In addition to using regularization techniques to improve generalizability, all models were fit with cross-validation. The purpose of cross-validation is to test the generalizability of a model within a sample. We report a very strong fit for our cross-validated models.
3. Replication in an additional sample: We demonstrate the generalizability of our findings by showing that the depths of sulci that are associated with reasoning in the Discovery sample generalize to the Replication sample. Our regularized regression reveals that the depths of a subset of RH tertiary sulci are relevant for reasoning performance in the Discovery sample (Fig. 4). We then show that these same sulci can be used to predict reasoning score with high accuracy in the Replication sample (Fig. 5).
4. Bootstrapped error estimates: We used bootstrapping as a diagnostic tool to assess the generalizability of our models to out-of-sample data. Using 10,000 iterations, we show our chosen models have low variance in estimated error (Fig. 5c), suggesting that they are not overfit to the data, and the findings will likely generalize to other samples.

#### Model selection—Discovery sample

We applied a least absolute shrinkage and selection operator (LASSO) regression model to determine which sulci, if any, were associated with Matrix reasoning. The depth of all 12 LPFC sulci was included as predictors in the regression model. LASSO performs L1 regularization by applying a penalty, or shrinking parameter (*α*), to the absolute magnitude of the coefficients such that:

$$({{{{{\rm{||}}}}}}y-x\beta {{{{{\rm{|}}}}}}{{{{{{\rm{|}}}}}}}_{2}^{2}+\alpha {{{{{\rm{||}}}}}}\beta {{{{{\rm{|}}}}}}{{{{{{\rm{|}}}}}}}_{1})\,$$

In a LASSO regression, low coefficients are set to zero and eliminated from the model. In this way, LASSO can facilitate variable selection, leading to simplified models with increased interpretability and prediction accuracy^69^. In our case, the LASSO regression algorithm shrinks the coefficients of each of the sulci until only the sulci most strongly associated with reasoning remain in the model. The LASSO regression model was conducted separately for the left and right hemispheres. By convention, we used cross-validation to select the shrinking parameter (*α*). We used the SciKit-learn GridSearchCV package^105^ to perform an exhaustive search across a range of *α*-values (0.01–10.0), and selected the value that minimized cross-validated mean-squared error (MSE_CV_).

#### Model evaluation—Replication sample

To further characterize the relationship between sulcal depth and reasoning performance, we used the predictors identified by the LASSO regression in the Discovery sample to predict Matrix reasoning score in the Replication Sample. As age is correlated with Matrix reasoning score, we included age as an additional covariate in the model (1). We fit this model as well as alternate nested models with leave-one-out cross-validation (looCV). We used nested model comparison to assess the unique variance explained by sulcal depth, while accounting for age-related effects on reasoning:

1 $${y}_{i}={{{{{\beta }}}}}_{0}+{{{{{\beta }}}}}_{1}{{{{{\rm{Age}}}}}}+{{{{{\beta }}}}}_{2}{{{{{\rm{pmfs}}}}}}\_{{{{{\rm{i}}}}}}+{{{{{\beta }}}}}_{3}{{{{{\rm{pmfs}}}}}}\_{{{{{\rm{a}}}}}}+{{{{{\beta }}}}}_{4}{{{{{\rm{pimf}}}}}}s+\in {{{{{\rm{I}}}}}}$$

In addition, we conducted this analysis with only the two most strongly associated sulci (pmfs-i, pimfs) from the Discovery sample:

2 $${y}_{i}={{{{{\beta }}}}}_{0}+{{{{{\beta }}}}}_{1}{{{{{\rm{Age}}}}}}+{{{{{\beta }}}}}_{2}{{{{{\rm{pmfs}}}}}}\_{{{{{\rm{i}}}}}}+{{{{{\beta }}}}}_{3}{{{{{\rm{pimfs}}}}}}+\in {{{{{\rm{I}}}}}}$$

To assess the unique variance explained by tertiary sulcal depth, we compared the MSE_CV_ of this model to the MSE_CV_ of a model with age as the sole predictor (3):

3 $${y}_{i}={{{{{\beta }}}}}_{0}+{{{{{\beta }}}}}_{1}{{{{{\rm{Age}}}}}}+\in {{{{{\rm{I}}}}}}$$

As these models are nested (all predictors in the smaller model (3) are also included in the larger models (1), (2)), we are able to directly compare the prediction error in these two models. Finally, to assess the specificity of the relationship to tertiary sulci in our Replication sample, we assessed the fit of model (1) to a full model that included all identified LPFC sulci within a hemisphere (4). The full model is as follows:

4 $${y}_{i}={{{{{\beta }}}}}_{0}+{{{{{\beta }}}}}_{1}{{{{{\rm{Age}}}}}}+{{{{{\beta }}}}}_{2\times 2}\ldots +{{{{{\beta }}}}}_{12\times 12}\in {{{{{\rm{I}}}}}}$$

where *x*_2_…*x*_12_ represent the sulcal depth of each identified sulcus within a hemisphere.

#### Empirical MSE confidence intervals

The size (*n* = 27) of the Replication sample makes looCV suitable. However, models that are fit with looCV can have high variance. Thus, to assess the potential variance in our estimations, we performed a bootstrapping procedure to empirically estimate the distribution of possible MSEcv predictions for models 1, 2, 3, and 4. For each model, data were randomly selected with replacement 10,000 times and MSEcv was computed for each iteration. From this process, we estimate Median MSE and 95% confidence intervals for each model (shown in Fig. 5c). All analyses were conducted with SciKit-Learn package in Python^105^.

### Assessing morphological and behavioral preference of the model

#### Cortical thickness

To assess whether our findings generalized to other anatomical features, we considered cortical thickness, which is an anatomical feature commonly explored in developmental cognitive neuroscience studies^73,97,106,107^. To do so, we replaced sulcal depth with cortical thickness as the predictive metric in our best-performing model in the Replication sample [Model 2]. As with depth, the model was fit to the data with looCV. To compare the thickness model to the depth model, we used the Akaike Information Criterion (AIC), which provides an estimate of in-sample prediction error and is suitable for non-nested model comparison. AIC is given by:

$${{{{{{\mathrm{AIC}}}}}}}_i=-2{\log }L_i+2K_i$$

where *L*_*i*_ is the likelihood for the model (*i*) and *K*_*i*_ is the number of parameters. By comparing AIC scores, we are able to assess the relative performance of the two models. If the ∆AIC is >2, it suggests an interpretable difference between models. If the ∆AIC is >10, it suggests a strong difference between models, with the lower AIC value indicating the preferred model^75,76^.

#### Processing speed and working memory

To ascertain whether the relationship between sulcal depth and cognition is specific to reasoning performance, or transferable to other general measures of cognitive processing^99^, we investigated the generalizability of the sulcal-behavior relationship to two other widely used measures of cognitive functioning: Processing speed and working memory. Specifically, we used looCV to predict processing speed (as indexed by Cross Out score) and working memory (as indexed by Digit Span Forwards score)^108^ instead of Matrix Reasoning score. In the cases in which the model showed a strong association, we used AIC to compare the model predictions to Matrix reasoning predictions.

### Reporting summary

Further information on research design is available in the Nature Research Reporting Summary linked to this article.

## Supplementary information

Editor Summary.

Supplementary Information.

Reporting Summary.

## Data Availability

The morphological metrics, behavioral data, and demographics have been made freely available under accession code 10.5281/zenodo.5021232^109^. The processed data required to perform all statistical analyses and to reproduce all figures are freely available at the above link. Probabilistic maps of all sulci aligned to the FreeSurfer average cortical surface (fsaverage) are also available for download. Visualizations of all sulcal definitions generated for each subject are provided in the Supplementary Information. Requests for further information or raw data should be directed to the Corresponding Author, Willa Voorhies (wvoorhies@berkeley.edu). Source data are provided with this paper.

## Source data

Source Data.

## Code availability

We provide all of the custom code to perform statistical analyses and to generate all figures on GitHub in an implementable binder notebook. We also provide the custom code used to generate sulcal probability maps in the same repository (https://github.com/cnl-berkeley/stable_projects/tree/main/CognitiveInsights_SulcalMorphology).

## References

[1] Zilles K, Palomero-Gallagher N, Amunts K (2013). Development of cortical folding during evolution and ontogeny. Trends Neurosci..

[2] Donahue CJ, Glasser MF, Preuss TM, Rilling JK, Van Essen DC (2018). Quantitative assessment of prefrontal cortex in humans relative to nonhuman primates. Proc. Natl Acad. Sci. USA.

[3] Fuster JM (2001). The prefrontal cortex—an update: time is of the essence. Neuron.

[4] Badre D, Nee DE (2018). Frontal cortex and the hierarchical control of behavior. Trends Cogn. Sci..

[5] Szczepanski SM, Knight RT (2014). Insights into human behavior from lesions to the prefrontal cortex. Neuron.

[6] Vendetti MS, Bunge SA (2014). Evolutionary and developmental changes in the lateral frontoparietal network: a little goes a long way for higher-level cognition. Neuron.

[7] Bailey, P. & Bonin, G. V. *The Isocortex of Man*. Vol. 1 (Illinois Monogr. Mediacal Sci., 1951).

[8] Bailey, P., Bonin, G. V. & McCulloch, W. S. *The Isocortex of the Chimpanzee.* Vol. 292 (Univ. Illinois Press, 1950).

[9] Sanides F (1964). Structure and function of the human frontal lobe. Neuropsychologia.

[10] Turner OA (1948). Growth and development of the cerebral cortical pattern in man. Arch. Neurol. Psychiatry.

[11] Weiner KS (2019). The Mid-Fusiform Sulcus (*sulcus sagittalis gyri fusiformis*). Anat. Rec..

[12] Weiner KS (2014). The mid-fusiform sulcus: a landmark identifying both cytoarchitectonic and functional divisions of human ventral temporal cortex. Neuroimage.

[13] Weiner KS, Zilles K (2016). The anatomical and functional specialization of the fusiform gyrus. Neuropsychologia.

[14] Welker W (1990). Why does cerebral cortex fissure and fold? A review determinants of gyri and sulci. Cereb. cortex.

[15] Tamraz, J. & Comair, Y. *Atlas of Regional Anatomy of the Brain Using MRI: with Functional Correlations.* (Springer-Verlag Berlin Heidelberg, 2006).

[16] Amiez C (2019). Sulcal organization in the medial frontal cortex provides insights into primate brain evolution. Nat. Commun..

[17] Amiez C (2021). Chimpanzee histology and functional brain imaging show that the paracingulate sulcus is not human-specific. Commun. Biol.

[18] Armstrong E, Schleicher A, Omran H, Curtis M, Zilles K (1995). The ontogeny of human gyrification. Cereb. Cortex.

[19] Chi JG, Dooling EC, Gilles FH (1977). Gyral development of the human brain. Ann. Neurol..

[20] Garrison JR (2015). Paracingulate sulcus morphology is associated with hallucinations in the human brain. Nat. Commun..

[21] Miller, J. A., D’esposito, M. & Weiner, K. S. Using tertiary sulci to map the ‘cognitive globe’ of prefrontal cortex. *J. Cogn. Neurosci*. 1–18 (2021). 10.1162/jocn_a_01696 34375416

[22] Connolly CJ (1940). Development of the cerebral sulci. Am. J. Phys. Anthropol..

[23] Connolly, C. J. *External Morphology of the Primate Brain.* (Springf. C. C. Thomas, 1950).

[24] Cunningham, D. *Contribution to the Surface Anatomy of the Cerebral Hemispheres.* (Dublin R. Irish Acad., 1892).

[25] Miller JA, Voorhies WI, Lurie DJ, D’Esposito M, Weiner KS (2021). Overlooked tertiary sulci serve as a meso-scale link between microstructural and functional properties of human lateral prefrontal cortex. J. Neurosci..

[26] Petrides, M. *Atlas of the Morphology of the Human Cerebral Cortex on the Average MNI Brain.* (Academic Press, 2019).

[27] Retzius, G. *Das menschenhirn. Studien in der makroskopischen morphologie.* (Norstedt, 1896).

[28] Sanides F (1962). Architectonics of the human frontal lobe of the brain. With a demonstration of the principles of its formation as a reflection of phylogenetic differentiation of the cerebral cortex. Monogr. Gesamtgeb. Neurol. Psychiatr..

[29] Weiner KS, Natu VS, Grill-Spector K (2018). On object selectivity and the anatomy of the human fusiform gyrus. NeuroImage.

[30] Miller JA (2020). Sulcal morphology of ventral temporal cortex is shared between humans and other hominoids. Sci. Rep..

[31] Wendelken C (2017). Frontoparietal structural connectivity in childhood predicts development of functional connectivity and reasoning ability: A large-scale longitudinal investigation. J. Neurosci..

[32] Penn DC, Holyoak KJ, Povinelli DJ (2008). Darwin’s mistake: explaining the discontinuity between human and nonhuman minds. Behav. Brain Sci..

[33] Buckner R (2012). The serendipitous discovery of the brain’s default network. Neuroimage.

[34] Rakic P (2009). Evolution of the neocortex: a perspective from developmental biology. Nat. Rev. Neurosci..

[35] Fry AF, Hale S (2000). Relationships among processing speed, working memory, and fluid intelligence in children. Biol. Psychol..

[36] Mcardle JJ, Ferrer-Caja E, Hamagami F, Woodcock RW (2002). Comparative longitudinal structural analyses of the growth and decline of multiple intellectual abilities over the life span. Dev. Psychol..

[37] Wendelken C, Ferrer E, Whitaker KJ, Bunge SA (2016). Fronto-parietal network reconfiguration supports the development of reasoning ability. Cereb. Cortex.

[38] Ferrer E (2013). White matter maturation supports the development of reasoning ability through its influence on processing speed. Dev. Sci..

[39] He W, Seymour RA (2018). Commentary: Frontoparietal structural connectivity in childhood predicts development of functional connectivity and reasoning ability: a large-scale longitudinal investigation. Front. Psychol..

[40] Crone EA, Wendelken C, Donohue S, Van Leijenhorst L, Bunge SA (2006). Neurocognitive development of the ability to manipulate information in working memory. Proc. Natl Acad. Sci. USA.

[41] Badre D, D’Esposito M (2009). Is the rostro-caudal axis of the frontal lobe hierarchical?. Nat. Rev. Neurosci..

[42] Christoff K (2001). Rostrolateral prefrontal cortex involvement in relational integration during reasoning. Neuroimage.

[43] Krawczyk DC, Michelle McClelland M, Donovan CM (2011). A hierarchy for relational reasoning in the prefrontal cortex. Cortex.

[44] Wendelken C, O’Hare ED, Whitaker KJ, Ferrer E, Bunge SA (2011). Increased functional selectivity over development in rostrolateral prefrontal. Cortex. J. Neurosci..

[45] Van Essen, D. C. *Cerebral Cortical Folding Patterns in Primates: Why They Vary and What They Signify.* Vol 4. 267–276 (Elsevier Inc., 2007).

[46] Lopez-Persem A, Verhagen L, Amiez C, Petrides M, Sallet J (2019). The human ventromedial prefrontal cortex: Sulcal morphology and its influence on functional organization. J. Neurosci..

[47] Dumontheil I, Houlton R, Christoff K, Blakemore SJ (2010). Development of relational reasoning during adolescence. Dev. Sci..

[48] Dumontheil I (2014). Development of abstract thinking during childhood and adolescence: the role of rostrolateral prefrontal cortex. Develop. Cogn. Neurosci..

[49] Blair C (2006). How similar are fluid cognition and general intelligence? A developmental neuroscience perspective on fluid cognition as an aspect of human cognitive ability. Behav. Brain Sci..

[50] Cattell, R. B. *Intelligence: Its Structure, Growth, and Action.* (North-Holland, 1987).

[51] Brun L (2016). Localized misfolding within Broca’s area as a distinctive feature of autistic disorder. Biol. Psychiatry Cogn. Neurosci. Neuroimaging.

[52] Amiez C, Petrides M (2018). Functional rostro-caudal gradient in the human posterior lateral frontal cortex. Brain Struct. Funct..

[53] Borne L, Rivière D, Mancip M, Mangin JF (2020). Automatic labeling of cortical sulci using patch- or CNN-based segmentation techniques combined with bottom-up geometric constraints. Med. Image Anal..

[54] Mangin, J. F. et al. in *Brain Mapping: An Encyclopedic Reference.* (Academic Press, 2015).

[55] Amiez C (2013). The location of feedback-related activity in the midcingulate cortex is predicted by local morphology. J. Neurosci..

[56] Amiez C, Petrides M (2007). Selective involvement of the mid-dorsolateral prefrontal cortex in the coding of the serial order of visual stimuli in working memory. Proc. Natl Acad. Sci. USA.

[57] Petrides M, Tomaiuolo F, Yeterian EH, Pandya DN (2012). The prefrontal cortex: comparative architectonic organization in the human and the macaque monkey brains. Cortex.

[58] Ariens-Kappers, C. *The Evolution of the Nervous System in Invertebrates, Vertebrates and Man.* (Haarlem Erven F. Bohn, 1929).

[59] Eberstaller, O. *Das Stirnhirn; ein Beitrag zur Anatomie der Oberfläche des Grosshirns.* (Wien Urban Schwarz, 1890).

[60] Ono, M., Kubik, S. & Abernathey, C. D. *Atlas of the Cerebral Sulci.* (New York Thieme Med. Publ. Inc., 1990).

[61] Rajkowska G, Goldman-Rakic PS (1995). Cytoarchitectonic definition of prefrontal areas in the normal human cortex: II. Variability in locations of areas 9 and 46 and relationship to the talairach coordinate system. Cereb. Cortex.

[62] Shellshear JL (1937). The brain of the Aboriginal Australian. A study in cerebral morphology. Philos. Trans. R. Soc. Lond. B. Biol. Sci..

[63] Petrides, M. *The Human Cerebral Cortex.* (Academic Press, 2012).

[64] Cachia A (2018). How interindividual differences in brain anatomy shape reading accuracy. Brain Struct. Funct..

[65] Cachia A (2015). Deviations in cortex sulcation associated with visual hallucinations in schizophrenia. Mol. Psychiatry.

[66] Dale AM, Fischl B, Sereno MI (1999). Cortical surface-based analysis I. Segmentation and surface reconstruction. NeuroImage.

[67] Fischl B, Dale AM, Raichle ME (2000). Measuring the thickness of the human cerebral cortex from magnetic resonance images. PNAS.

[68] Ghojogh, B. & Crowley, M. The theory behind overfitting, cross validation, regularization, bagging, and boosting: tutorial. Preprint at https://arxiv.org/abs/1905.12787 (2019).

[69] Heinze G, Wallisch C, Dunkler D (2018). Variable selection—A review and recommendations for the practicing statistician. Biometrical J..

[70] Burgaleta M, Johnson W, Waber DP, Colom R, Karama S (2014). Cognitive ability changes and dynamics of cortical thickness development in healthy children and adolescents. Neuroimage.

[71] Dickerson BC (2008). Detection of cortical thickness correlates of cognitive performance: Reliability across MRI scan sessions, scanners, and field strengths. Neuroimage.

[72] Østby Y (2009). Heterogeneity in subcortical brain development: a structural magnetic resonance imaging study of brain maturation from 8 to 30 years. J. Neurosci..

[73] Gogtay N (2004). Dynamic mapping of human cortical development during childhood through early adulthood. Proc. Natl Acad. Sci. USA.

[74] Kail R, Salthouse TA (1994). Processing speed as a mental capacity. Acta Psychol..

[75] Wagenmakers E-J, Farrell S, Wagenmakers J (2004). AIC model selection using Akaike weights. Psychnomic Bull. Rev..

[76] Burnham KP, Anderson DR (2004). Multimodel inference: understanding AIC and BIC in model selection. Sociological Methods Res..

[77] Kail RV, Lervåg A, Hulme C (2016). Longitudinal evidence linking processing speed to the development of reasoning. Dev. Sci..

[78] Holyoak KJ, Monti MM (2021). Relational integration in the human brain: a review and synthesis. J. Cogn. Neurosci..

[79] Rogers J (2010). On the genetic architecture of cortical folding and brain volume in primates. Neuroimage.

[80] Leroy F (2015). New human-specific brain landmark: the depth asymmetry of superior temporal sulcus. Proc. Natl Acad. Sci. USA.

[81] Im K (2010). Spatial distribution of deep sulcal landmarks and hemispherical asymmetry on the cortical surface. Cereb. Cortex.

[82] Liu T (2011). The relationship between cortical sulcal variability and cognitive performance in the elderly. Neuroimage.

[83] Borst G (2014). Folding of the anterior cingulate cortex partially explains inhibitory control during childhood: a longitudinal study. Dev. Cogn. Neurosci..

[84] Reveley C (2015). Superficial white matter fiber systems impede detection of long-range cortical connections in diffusion MR tractography. Proc. Natl Acad. Sci. USA.

[85] Ehsan SK, Motes MA, Rypma B, Krawczyk DC (2012). The network architecture of cortical processing in visuo-spatial reasoning. Sci. Rep..

[86] Turner R (2019). Myelin and modeling: Bootstrapping cortical microcircuits. Front. Neural Circuits.

[87] Van Essen DC (1997). A tension-based theory of morphogenesis and compact wiring in the central nervous system. Nature.

[88] Oyefiade AA (2018). Development of short-range white matter in healthy children and adolescents. Hum. Brain Mapp..

[89] Natu VS (2021). Sulcal depth in medial ventral temporal cortex predicts the location of a place-selective region in macaques, children, and adults. Cereb. Cortex.

[90] Watson CE, Chatterjee A (2012). A bilateral frontoparietal network underlies visuospatial analogical reasoning. Neuroimage.

[91] Van Essen DC, Donahue CJ, Glasser MF (2018). Development and evolution of cerebral and cerebellar cortex. Brain. Behav. Evol..

[92] Lebel C, Beaulieu C (2011). Longitudinal development of human brain wiring continues from childhood into adulthood. J. Neurosci..

[93] Lebel C, Treit S, Beaulieu C (2017). A review of diffusion MRI of typical white matter development from early childhood to young adulthood. NMR Biomed..

[94] Marek, S. et al. Towards reproducible brain-wide association studies. Preprint at *bioRxiv*10.1101/2020.08.21.257758 (2020).

[95] Lyu I (2021). Labeling lateral prefrontal sulci using spherical data augmentation and context-aware training. Neuroimage.

[96] Wechsler, D. *Wechsler Intelligence Scale for Children–Fourth Edition (WISC-IV)* (The Psychological Corporation, 1949).

[97] Brown TT (2012). Neuroanatomical assessment of biological maturity. Curr. Biol..

[98] McBride-Chang C, Kail RV (2002). Cross-cultural similarities in the predictors of reading acquisition. Child Dev..

[99] Kail RV, Ferrer E (2007). Processing speed in childhood and adolescence: longitudinal models for examining developmental change. Child Dev..

[100] Wandell BA, Chial S, Backus BT (2000). Visualization and measurement of the cortical surface. J. Cogn. Neurosci..

[101] Lüsebrink F, Sciarra A, Mattern H, Yakupov R, Speck O (2017). T1-weighted in vivo human whole brain MRI dataset with an ultrahigh isotropic resolution of 250 μm. Sci. Data.

[102] Edlow BL (2019). 7 Tesla MRI of the ex vivo human brain at 100 micron resolution. Sci. Data.

[103] Chen CH (2012). Hierarchical genetic organization of human cortical surface area. Science.

[104] Champod AS, Petrides M (2007). Dissociable roles of the posterior parietal and the prefrontal cortex in manipulation and monitoring processes. PNAS.

[105] Pedregosa F (2011). Scikit-learn: machine learning in python. J. Mach. Learn. Res..

[106] Tamnes CK (2013). Longitudinal working memory development is related to structural maturation of frontal and parietal cortices. J. Cogn. Neurosci..

[107] Vijayakumar N (2014). Thinning of the lateral prefrontal cortex during adolescence predicts emotion regulation in females. Soc. Cogn. Affect. Neurosci..

[108] Woodcock, R., Mather, N., McGrew, K. & Wendling, B. *Woodcock-Johnson III Tests of Cognitive Abilities* (Riverside Publishing Company, 2001).

[109] Voorhies, W. I. & Miller, J. A. CognitiveInsights_SulcalMorpholgy. cnl-berkeley/stable_projects-v1.1.0. 10.5281/zenodo.5021232 (2021).

