# Supplementary material for: Cognitive insights from tertiary sulci in prefrontal cortex
Source: Nat Commun. 2021 Aug 25;12:5122. doi: 10.1038/s41467-021-25162-w (PMC8387420; doi:10.1038/s41467-021-25162-w)
Supplement: Supplementary file 1 — Editor Summary. [file 41467_2021_25162_MOESM1_ESM.docx]

Tertiary sulci are shallow cortical folds that emerge late in gestation. Here the authors link prefrontal tertiary sulcal depth with reasoning scores in children and adolescents.
