# Supplementary material for: Cognitive insights from tertiary sulci in prefrontal cortex
Source: Nat Commun. 2021 Aug 25;12:5122. doi: 10.1038/s41467-021-25162-w (PMC8387420; doi:10.1038/s41467-021-25162-w)
Supplement: Supplementary file 2 — Supplementary Information. [file 41467_2021_25162_MOESM2_ESM.pdf]

Supplementary Information

Tertiary sulci in non-human primates

It is widely accepted that the cerebral cortices of humans and non-human hominoids have tertiary sulci, while non-human primates (NHPs) have shallow dimples, some of which are proposed to be homologous to tertiary sulci[1–8]. Immediately relevant for the present paper are proposals hypothesizing that PFC dimples in NHPs have deepened through the course of evolution to form tertiary sulci in PFC[6,7].

Developmental timeline of LPFC sulci

Broadly, primary, secondary, and tertiary sulci are defined based on their emergence in gestation [1–4,6,8–11]. The classification of sulci in this study is based on the following quotations from classic anatomical literature:

"The lateral fissure, s. cinguli, s. olfactorius, s. calcarinus, and s. parieto-occipitalis became visible in OW 16. The s. centralis and s. temporalis superior appeared during OW 21. Between OW 25 and 28, a time of rapid growth of the cortex, the following sulci became recognizable: circularis insulae, fron- talis superior, intraparietalis, precentralis and orbitales. In OW 29 the s. postcentralis, s. frontalis inferior, and s. temporalis inferior became evident. Because these sulci appear first in development, they are termed primary sulci[10]. The secondary sulci, those that appear later and have a moderate degree of individual variability in shape and ori- entation, were found beginning in OW 32. At that time s. tem- poralis transversis became identifiable. The highly variable and late-appearing tertiary sulci[10] began to form in OW36." (pg. 57)[2]

"The sulci on the lateral surface may be seen as faint dimples or shallow furrows at the end of the fifth or beginning of the sixth month. During the sixth they develop further, and at the middle of the seventh, all the principal sulci are laid down usually in a simple form. In the eighth month the sulci become branched and tortuous and secondary sulci become marked. In the last month of fetal life the brain becomes rich in furrows with many secondary and tertiary sulci as if a miniature adult and at birth the insula may be almost completely covered."(pgs. 132-133)[11].

No differences in cortical thickness among primary and tertiary sulci in LPFC

As discussed throughout this paper, sulcal depth is the main morphological feature differentiating tertiary from primary and secondary sulci[1,4,9,10,12–15]. Nevertheless, previous

developmental work on structural variability in PFC has frequently focused on cortical thickness[16–19]. Thus, we also investigated variability in cortical thickness in both the Discovery and Replication samples as a function of sulcal type (tertiary vs. primary) and hemisphere (left vs. right). There were no significant differences in cortical thickness between primary and tertiary sulci in the Discovery ($F(1,27) = 2.44$, $p = 0.13$; $Mean(sd))_{Tertiary} = 2.41(0.36)$; $Mean(sd)_{Primary} = 2.37\ (0.26)$; Supplementary Fig. 3a) or Replication ($F(1,26) = 2.31$, $p =0.14$; $Mean(sd))_{Tertiary} = 2.31(0.41)$, $Mean(sd)_{Primary} = 2.38(0.30)$; Supplementary Fig. 3a) samples. The rm-ANOVA revealed a main effect of hemisphere in both samples in which right hemisphere sulci were cortically thinner than left hemisphere sulci (Discovery: ($F(1,27) = 123.1$, $p<10^{-3}$, $\eta^2_G = 0.09$; $Mean(sd)_{RH}; = 2.30\ (0.28)$; $Mean(sd)_{LH} = 2.47(0.27)$; Replication: ($F(1, 26) = 42.91$, $p<10^{-3,}$ $\eta^2_G= 0.06$; $Mean(sd)_{RH} = 2.20(0.36)$, $Mean(sd)_{LH} =2.41(0.29)$; Supplementary Fig. 3). Thus, while previous developmental work on structural variability in PFC has focused on cortical thickness[16–19], when considering tertiary sulci, the present analyses emphasize the utility of sulcal depth, not cortical thickness, for differentiating tertiary from primary sulci.

a

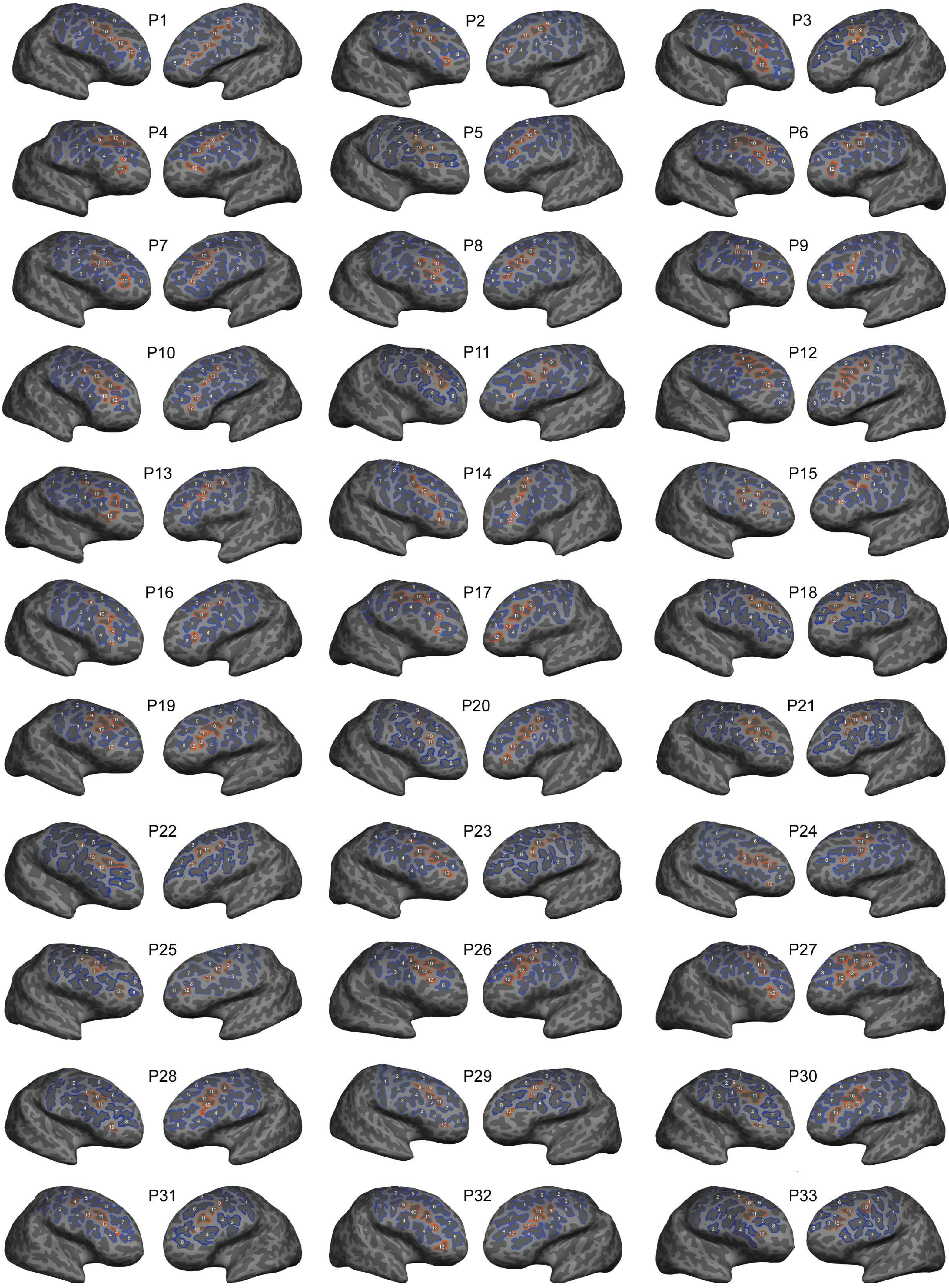
P1
P2
P3
P4
P5
P6
P7
P8
P9
P10
P11
P12
P13
P14
P15
P16
P17
P18
P19
P20
P21
P22
P23
P24
P25
P26
P27
P28
P29
P30
P31
P32
P33

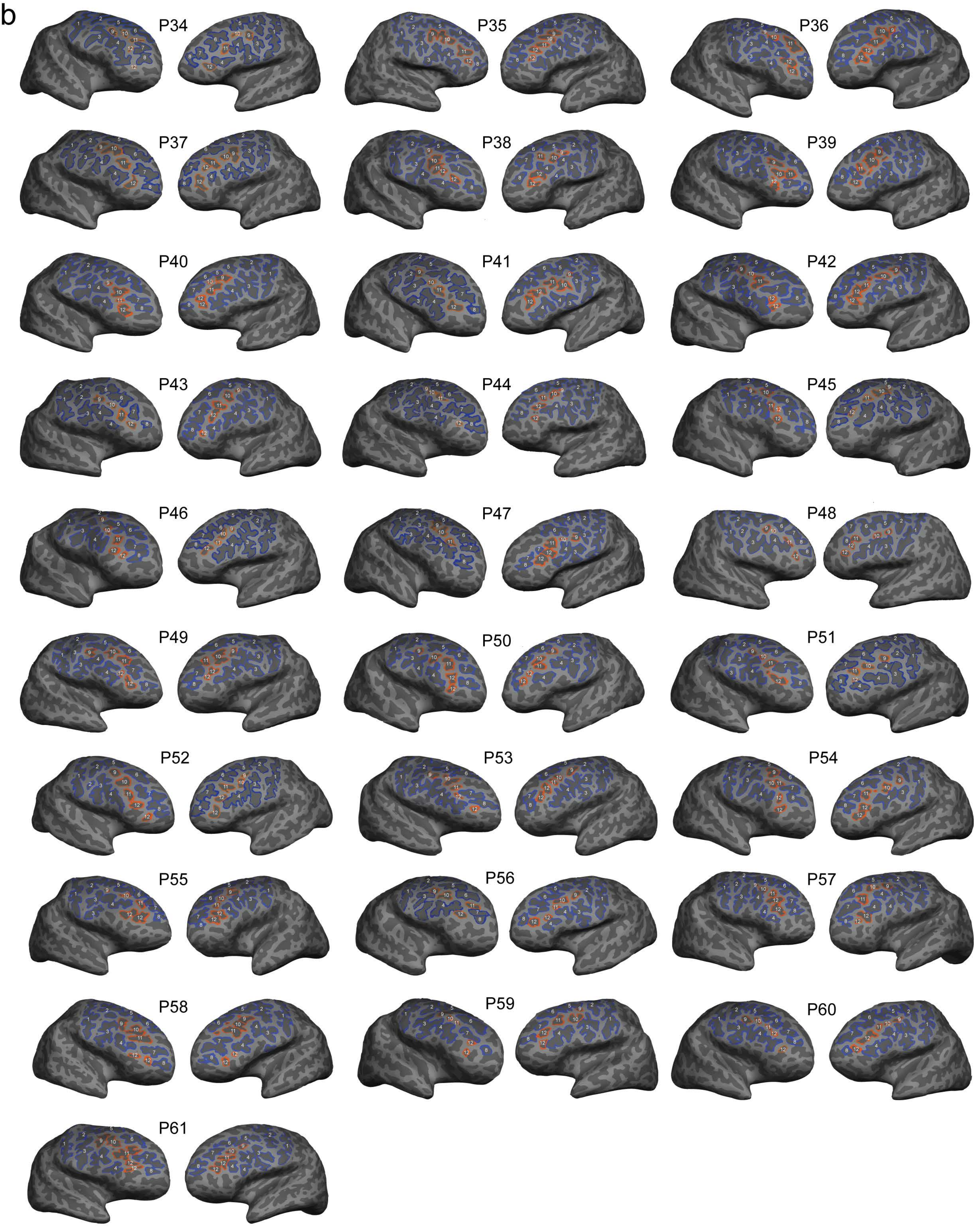

Supplementary Fig. 1. Manual labels in the left and right hemispheres of every participant displayed on the inflated cortical surface in FreeSurfer 6.0.0. We defined sulci on the inflated and pial cortical surfaces of each hemisphere for each participant. a. Manually labeled sulci on the inflated cortical surface in the left and right hemisphere for every participant in the Discovery Sample. Tertiary (orange) and primary (blue) sulci are identifiable in every participant. The pimfs (*) can contain two components, one component, or can be absent altogether in a given hemisphere (Supplementary Table 1). b. Same layout as in a., but for the Replication Sample. As in the Discovery Sample, tertiary (orange) and primary (blue) sulci are identifiable in every participant in which the pimfs could contain 0, 1, or 2 components. When present, the pimfs is indicated by *.

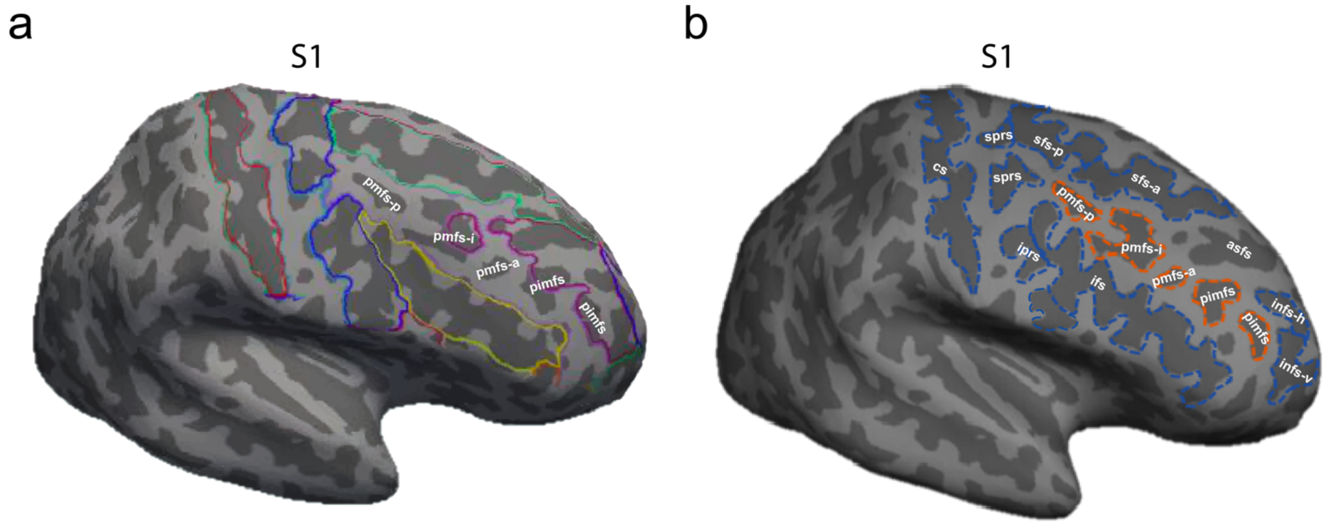

Supplementary Fig. 2. ***Lateral prefrotnal tertiary sulci are often omitted in commonly used atlases.*** **a.** Example inflated cortical surface reconstruction of a right hemisphere. Colors indicate sulcal and gyral definitions provided by automated methods[20,21]. The omitted tertiary sulci explored in the present study are labeled by the white acronyms. While the automated approach is useful for many studies, we manually defined sulci for our study as present approaches do not yet include tertiary sulci (labeled in white: pmfs-p, pmfs-i, pmfs-a, pimfs), and automated methods often include gyral components in the sulcal definitions. Red: central sulcus. Blue: pre-central sulcus. Yellow: inferior frontal sulcus. Turquoise: superior frontal sulcus. Magenta: fronto-marginal sulcus (which includes the horizontal and ventral components of the intermediate frontal sulcus, as well as portions of the pmfs-i, and what Petrides[26] refers to as the accessory superior frontal sulcus (asfs; not examined in the present study). **b.** Example of manual sulcal definitions in the same participant as in **a**. Manual definitions capture both tertiary (orange) and primary (blue) sulci.

	Discovery (n = 33)		Replication (n = 28)	
Components	***left***	***right***	***left***	***right***
0	2/33	3/33	1/28	0/28
1	15/33	18/33	3/28	9/28
2	16/33	12/33	24/28	19/28

Supplementary Table 1. ***Variability in the number of pimfs components across individuals.*** Participants had 0, 1, or 2 pimfs components in each hemisphere. As a majority of participants in both samples had at least one pimfs component, our inclusion criteria was to include participants who had at least one pimfs component in each hemisphere (Discovery: 28/33, Replication: 27/28), which assures that all repeated measures statistics are balanced for effects of sulcus and hemisphere. Source data are provided as a Source Data file.

Annual Household Income	Count
$16,000 to $24,999	2
$50,000 to $74,999	5
$75,000 to $99,999	7
Over $100,000	8
Over $200,000	3
Unknown/Not Reported	14
Highest Degree Earned by Parent/Guardian	
High School/ GED	7
Associate degree	6
Bachelor's degree	12
Master's degree	14
Doctorate	3
Professional	1
Other	2
None of the above (less than high school)	1
Ethnic Categories	
Hispanic or Latino	6
Not Hispanic or Latino	24
Unknown/ Not Reported	25
Racial Categories	
American Indian/Alaska Native	0
Asian	1
Native Hawaiian or Other Pacific Islander	0
Black or African American	3
White	31
More Than One Race	12
Unknown/Not Reported	8

Supplementary Table 2. ***Summary of sample demographics***. Parent/Guardian reported Race, Ethnicity, Family income, and Education are summarized across the Discovery and Replication samples for all participants included in the behavioral portion of the present study (N = 55). Source data are provided as a Source Data file.

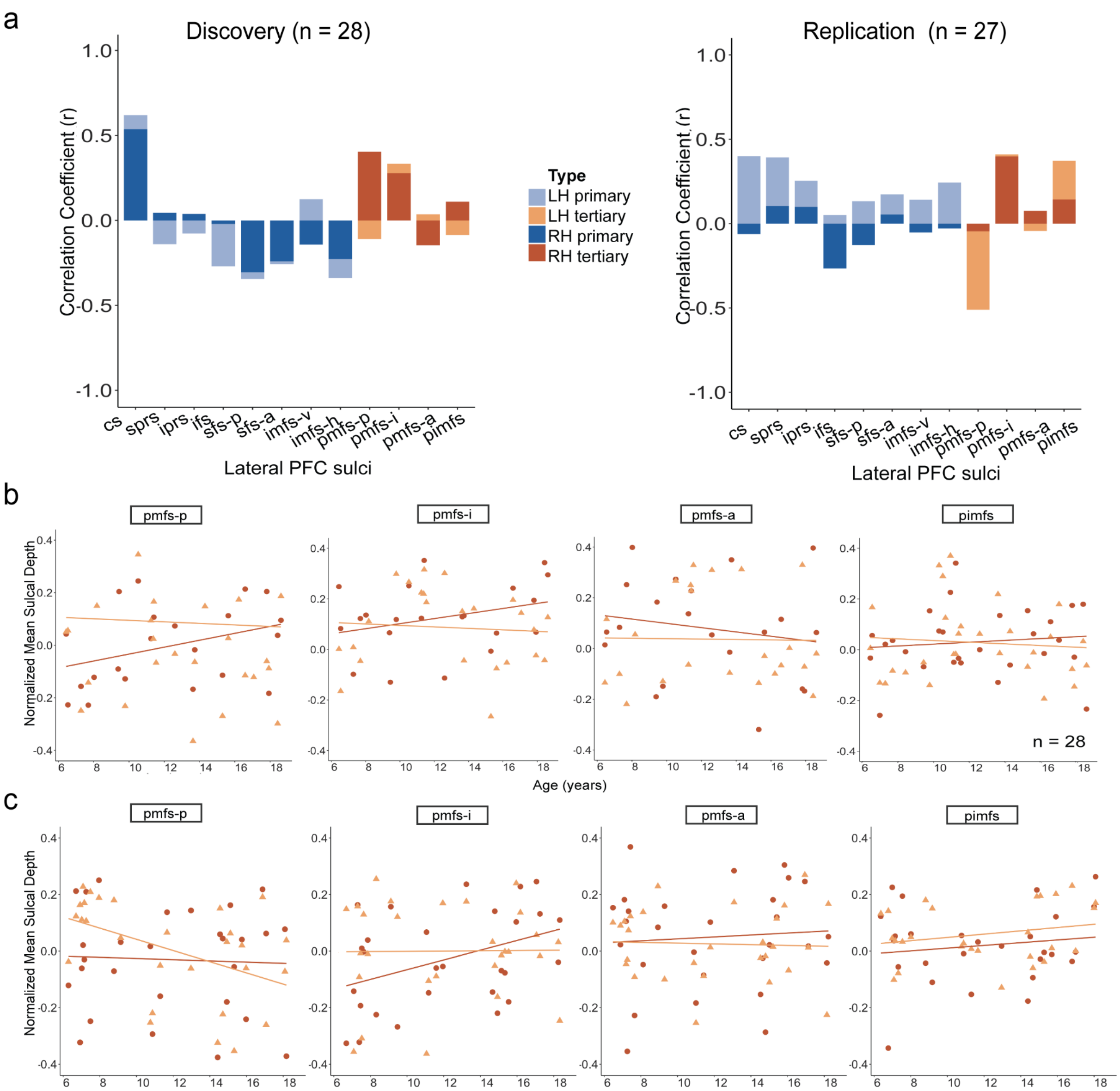

Supplementary Fig. 3. ***Morphological and behavioral associations with age in both Discovery and Replication samples.*** **a.** Correlation between age and sulcal depth in the Discovery (left) and Replication (right) samples. Each bar represents the correlation coefficient (Pearson's *r*) between sulcal depth and age for each sulcus (orange: tertiary; blue: primary) in the left (lighter shades) and right (darker shades) hemispheres. There is not a clear relationship between sulcal depth and age that is generalizable among LPFC sulci. **b-c.** Scatterplots showing the association between age and sulcal depth for each of the 4 tertiary sulci explored in the present study in each hemisphere (left: lighter triangles; right: darker circles) for individual participants in the Discovery (**b**) and Replication (**c**) samples. Age does not account well for individual variability in LPFC tertiary sulcal depth. Source data are provided as a Source Data file.

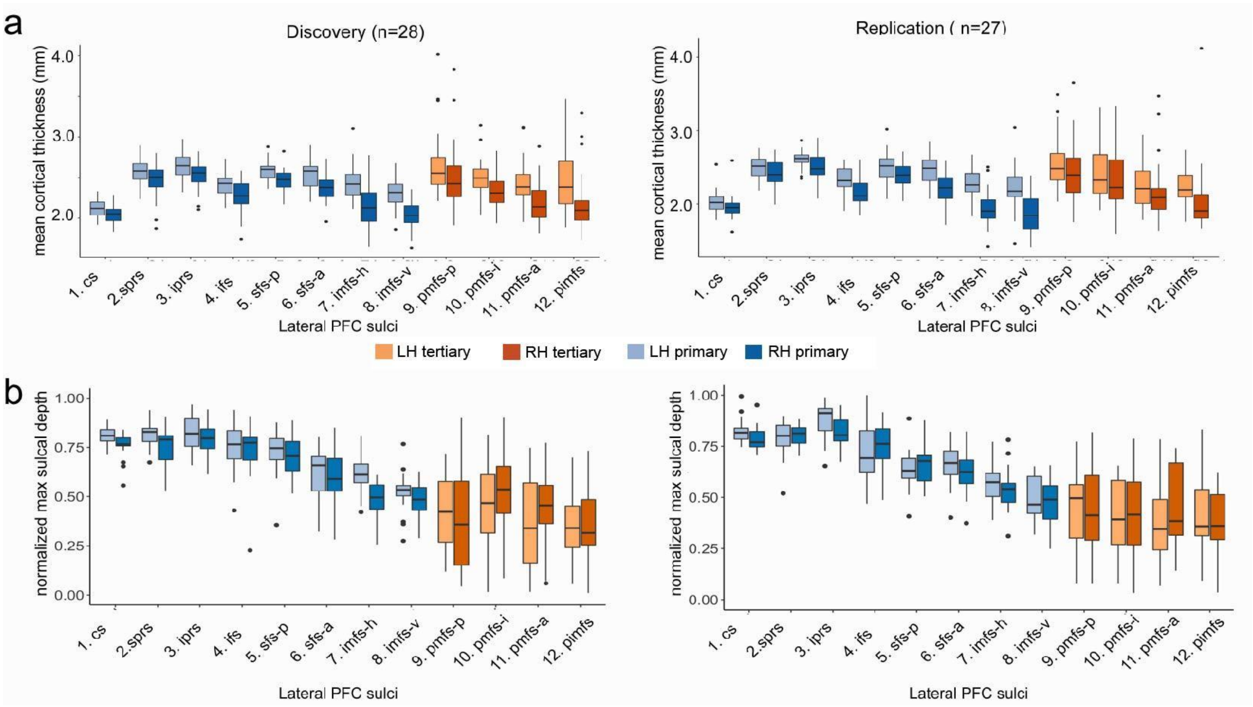

Supplementary Fig. 4. ***No difference in cortical thickness between tertiary and primary sulci in lateral prefrontal cortex*****.** **a.** *Left:* Mean cortical thickness for each of the 12 lateral prefrontal (LPFC) sulci in the Discovery sample (n = 28 participants). Right: Mean cortical thickness for each of the 12 LPFC sulci in the Replication sample (n = 27 participants). Tertiary sulci (orange) and primary sulci (blue) do not significantly differ in cortical thickness in either sample. **b.** Left: Maximum sulcal depth for each of the 12 LPFC sulci in the Discovery sample. Right: Maximum sulcal depth for each of the 12 LPFC sulci in the Replication sample. Depth was normalized by the deepest point in the individual's hemisphere. Light colors indicate left hemisphere (LH) sulci and dark colors indicate right hemisphere (RH) sulci respectively.Horizontal lines represent median values and whisker lines represent the 1st and 3rd quartiles. Values falling outside of this range are represented as black dots. Source data are provided as a Source Data file.

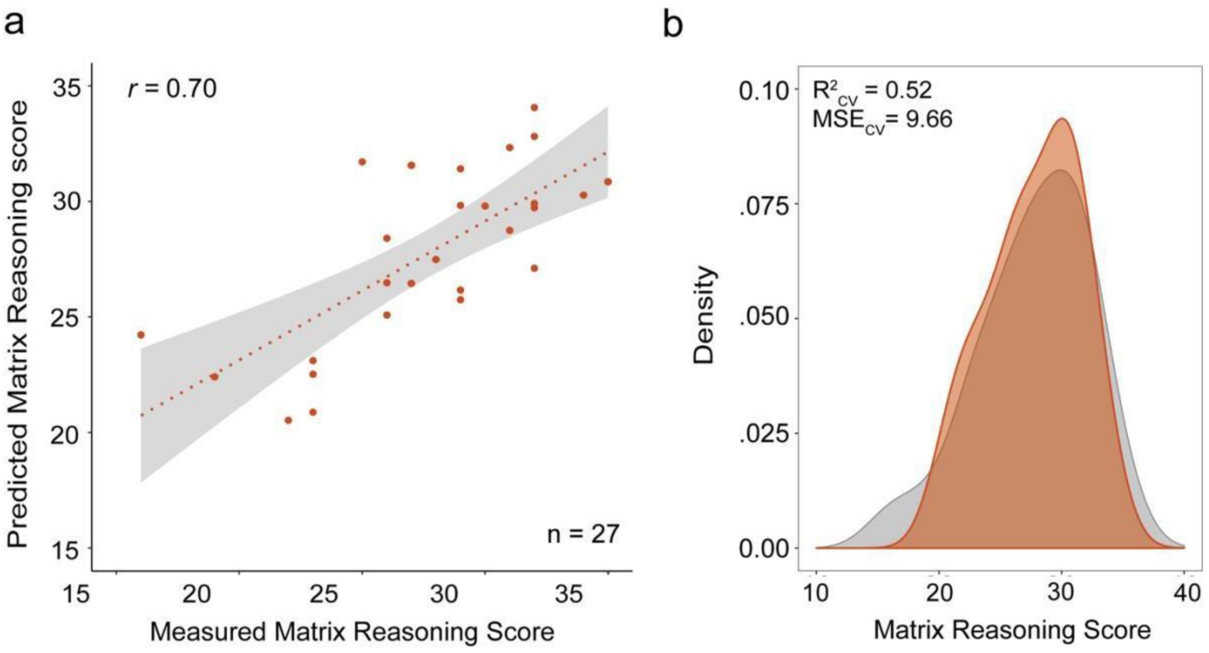

Supplementary Fig. 5. ***Predicted matrix reasoning score in the Replication sample from three tertiary sulci (pmfs-i, pmfs-a, pimfs).*** **a.** Spearman's correlation *(r)* between measured and predicted Matrix reasoning scores in the Replication sample for the model including all three tertiary sulci identified in the Discovery sample (pmfs-i_{RH}, pmfs- $a_{RH,}$ $pimfs_{RH}$). Gray bar represents the 95% confidence interval for the linear model. **b**. Density plot showing model fit indexed by cross-validated mean-squared error (MSE_{CV}) and model fit (R^2_{CV}). orange: The distribution of predicted scores from this model. gray: the distribution of measured Matrix reasoning scores. Source data are provided as a Source Data file.

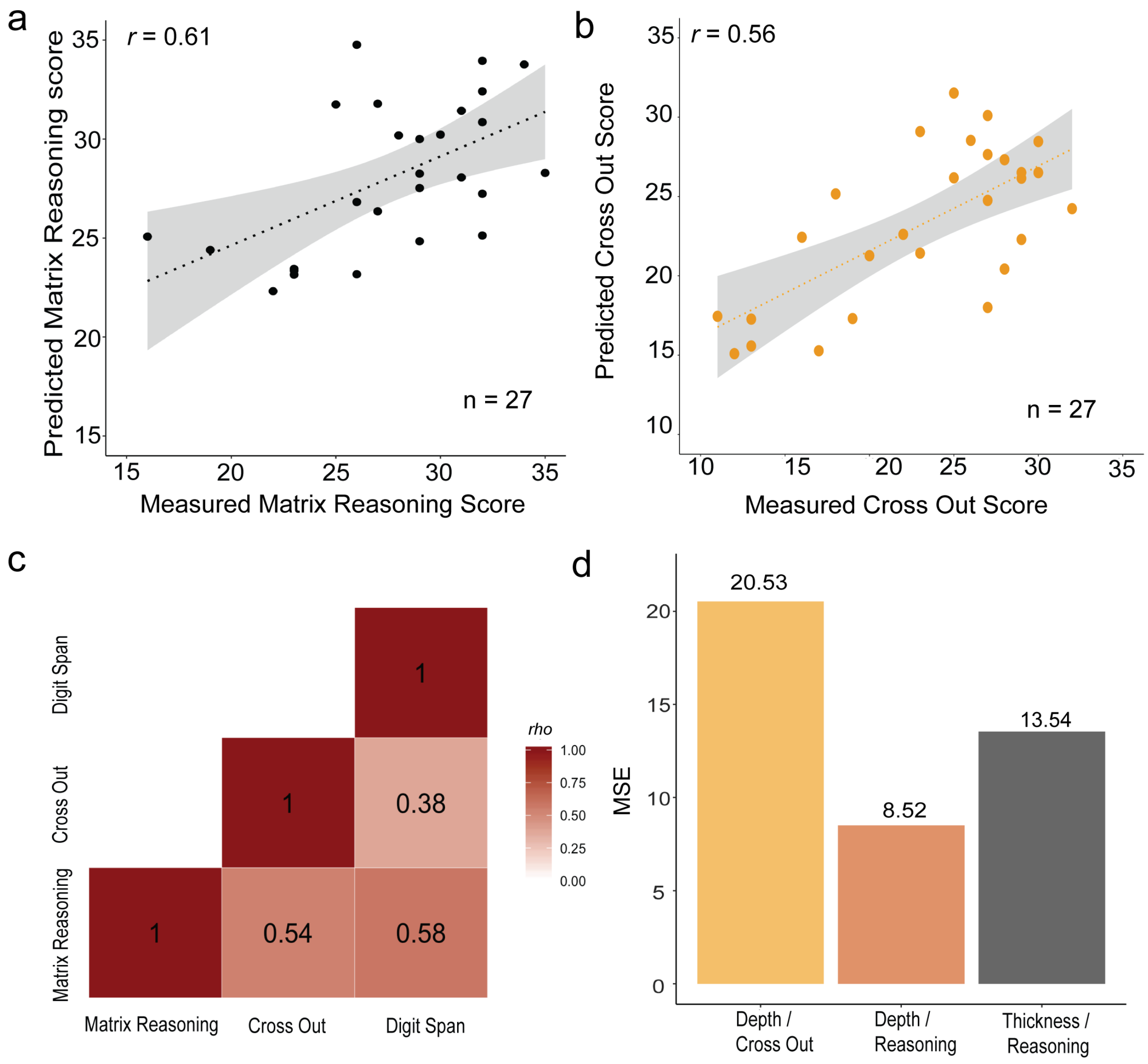

Supplementary Fig. 6. ***Tertiary sulcal depth more strongly relates to reasoning than cortical thickness and this relationship shows behavioral preference over other cognitive measures.*** **a.** Thickness was used in place of depth to predict Matrix reasoning in the Replication sample. The model was fit with looCV. Spearman's correlation (*r)* between measured and predicted Matrix reasoning scores in the Replication sample for the best model (pmfs-i_{RH} + $pimfs_{RH}$. + age). Gray bar represents the 95% confidence interval for the linear model. **b.** The same depth model was used to predict Cross Out score instead of Matrix reasoning in the Replication sample. Spearman's correlation between measured and predicted Cross Out scores in the Replication sample using the best performing depth model (pmfs-i_{RH} + $pimfs_{RH}$ + age). **c.** Correlation (Spearman's rho) between Matrix reasoning, Cross Out (Processing speed), and Digit Span (Working memory). Digit Span was included as an additional comparison measure in the Replication sample; it was not predicted by the depth model (R^2_{cv} = 0.10) and was not explored further. **d**. MSE_{cv} for the thickness and Cross Out score models compared to the analogous depth model. Tertiary sulcal depth offered substantially better predictions than cortical thickness. The depth model did not generalize to processing speed. Source data are provided as a Source Data file.

References

1. Welker, W. Why does cerebral cortex fissure and fold? A review determinants of gyri and sulci. *Cereb. cortex* **8b**, 3--136 (1990).
2. Armstrong, E., Schleicher, A., Omran, H., Curtis, M. & Zilles, K. The ontogeny of human gyrification. *Cereb. Cortex* **5**, 56–63 (1995).
3. Sanides, F. Architectonics of the human frontal lobe of the brain. With a demonstration of the principles of its formation as a reflection of phylogenetic differentiation of the cerebral cortex. *Monogr. Gesamtgeb. Neurol. Psychiatr.* **98**, 1–201 (1962).
4. Sanides, F. Structure and function of the human frontal lobe. *Neuropsychologia* **2**, 209–219 (1964).
5. Miller, J. A. *et al.* Sulcal morphology of ventral temporal cortex is shared between humans and other hominoids. *Sci. Rep.* **10**, 17132 (2020).
6. Miller, Jacob A. Mark D'esposito, Weiner, K.S. Using tertiary sulci to map the 'cognitive globe' of prefrontal cortex. *J. Cogn. Neurosci* **in press**, (2021).
7. Schall JD, Zinke W, Cosman JD, Schall MS, Pare M, P. P. On the Evolution of the frontal eye field: comparisons of monkeys, apes, and humans. in *Evolutionary Neuroscience, Ed 2* 861–883 (2020).
8. Amiez, C. *et al.* Sulcal organization in the medial frontal cortex provides insights into primate brain evolution. *Nat. Commun.* **10**, 3437 (2019).
9. Miller, J. A., Voorhies, W. I., Lurie, D. J., D'Esposito, M. & Weiner, K. S. Overlooked tertiary sulci serve as a meso-scale link between microstructural and functional properties of human lateral prefrontal cortex. *J. Neurosci.* **41**, 2229-2244 (2021).
10. Chi, J. G., Dooling, E. C. & Gilles, F. H. Gyral development of the human brain. *Ann. Neurol.* **1**, 86–93 (1977).
11. Connolly, C. J. Development of the cerebral sulci. *Am. J. Phys. Anthropol.* **26**, 113–149 (1940).
12. Weiner, K. S. The Mid‐Fusiform Sulcus (*sulcus sagittalis gyri fusiformis*). *Anat. Rec.* **302**, 1491–1503 (2019).
13. Weiner, K. S., Natu, V. S. & Grill-Spector, K. On object selectivity and the anatomy of the human fusiform gyrus. (2018) doi:10.1016/j.neuroimage.2018.02.040.
14. Petrides, M. *Atlas of the morphology of the human cerebral cortex on the average MNI brain*. (Academic Press, 2019).
15. Zilles, K., Armstrong, E., Schleicher, A. & Kretschmann, H.-J. The human pattern of gyrification in the cerebral cortex. *Anat. Embryol. (Berl).* **179**, 173–179 (1988).
16. Gogtay, N. *et al.* Dynamic mapping of human cortical development during childhood through early adulthood. *Proc. Natl. Acad. Sci. U. S. A.* **101**, 8174–8179 (2004).
17. Tamnes, C. K. *et al.* Longitudinal working memory development is related to structural maturation of frontal and parietal cortices. *J. Cogn. Neurosci.* **25**, 1611–1623 (2013).
18. Brown, T. T. *et al.* Neuroanatomical assessment of biological maturity. *Curr. Biol.* **22**, 1693–1698 (2012).
19. Vijayakumar, N. *et al.* Thinning of the lateral prefrontal cortex during adolescence predicts emotion regulation in females. *Soc. Cogn. Affect. Neurosci.* **9**, 1845–1854 (2014).
20. Dale, A. M., Fischl, B. & Sereno, M. I. Cortical Surface-Based Analysis I. Segmentation and Surface Reconstruction. *NeuroImage* **9**, 179-194 (1999).
21. Destrieux, C., Fischl, B., Dale, A. & Halgren, E. Automatic parcellation of human cortical gyri and sulci using standard anatomical nomenclature. *Neuroimage* (2010) doi:10.1016/j.neuroimage.2010.06.010.
