# Supplementary material for: Cognitive insights from tertiary sulci in prefrontal cortex
Source: Nat Commun. 2021 Aug 25;12:5122. doi: 10.1038/s41467-021-25162-w (PMC8387420; doi:10.1038/s41467-021-25162-w)
Supplement: Supplementary file 3 — Reporting Summary. [file 41467_2021_25162_MOESM3_ESM.pdf]

nature research

Corresponding author(s): Willa Voorhies

Last updated by author(s): 06/22/2021

Reporting Summary

Nature Research wishes to improve the reproducibility of the work that we publish. This form provides structure for consistency and transparency in reporting. For further information on Nature Research policies, see our Editorial Policies and the Editorial Policy Checklist.

Statistics

For all statistical analyses, confirm that the following items are present in the figure legend, table legend, main text, or Methods section.

n/a	Confirmed	
☐	☒	The exact sample size (*n*) for each experimental group/condition, given as a discrete number and unit of measurement
☐	☒	A statement on whether measurements were taken from distinct samples or whether the same sample was measured repeatedly
☐	☒	The statistical test(s) used AND whether they are one- or two-sided<br>*Only common tests should be described solely by name; describe more complex techniques in the Methods section.*
☐	☒	A description of all covariates tested
☐	☒	A description of any assumptions or corrections, such as tests of normality and adjustment for multiple comparisons
☐	☒	A full description of the statistical parameters including central tendency (e.g. means) or other basic estimates (e.g. regression coefficient) AND variation (e.g. standard deviation) or associated estimates of uncertainty (e.g. confidence intervals)
☐	☒	For null hypothesis testing, the test statistic (e.g. F, t, r) with confidence intervals, effect sizes, degrees of freedom and P value noted<br>*Give P values as exact values whenever suitable.*
☒	☐	For Bayesian analysis, information on the choice of priors and Markov chain Monte Carlo settings
☒	☐	For hierarchical and complex designs, identification of the appropriate level for tests and full reporting of outcomes
☐	☒	Estimates of effect sizes (e.g. Cohen's d, Pearson's r), indicating how they were calculated

Our web collection on statistics for biologists contains articles on many of the points above.

Software and code

Policy information about availability of computer code

Data collection	No software was used in data collection
Data analysis	MRI image processing and anatomical labeling was performed with the open source software: freesurfer 6.0.0 (https://surfer.nmr.mgh.harvard.edu/). All subsequent analyses were performed using custom Python (3.7.4) and R (3.6.2) code. The full data analysis pipeline, code, and corresponding data is freely available and executable on GitHub (https://github.com/wvoorhies/CognitiveInsights_SulcalMorphology). We have also obtained a DOI for this repository (DOI:10.5281/zenodo.5016092).

For manuscripts utilizing custom algorithms or software that are central to the research but not yet described in published literature, software must be made available to editors and reviewers. We strongly encourage code deposition in a community repository (e.g. GitHub). See the Nature Research guidelines for submitting code & software for further information.

Data

Policy information about availability of data

All manuscripts must include a data availability statement. This statement should provide the following information, where applicable:

- Accession codes, unique identifiers, or web links for publicly available datasets
- A list of figures that have associated raw data
- A description of any restrictions on data availability

The morphological metrics, behavioral data, and demographics have been made freely available under accession code DOI:10.5281/zendo.5016092 (https://zenodo.org/record/5016092#.YNJ_jzZKjX0). The processed data required to perform all statistical analyses and to reproduce all figures are freely available at the above link. Probabilistic maps of all sulci aligned to the FreeSurfer average cortical surface (fsaverage) are also available for download. Visualizations of all sulcal definitions generated for each subject are provided in the Supplementary Information. Source data to generate relevant figures are provided with this paper. Any requests for further information or raw data should be directed to the Corresponding Author, Willa Voorhies (wvoorhies@berkeley.edu).

Field-specific reporting

Please select the one below that is the best fit for your research. If you are not sure, read the appropriate sections before making your selection.

☒ Life sciences ☐ Behavioural & social sciences ☐ Ecological, evolutionary & environmental sciences

For a reference copy of the document with all sections, see nature.com/documents/nr-reporting-summary-flat.pdf

Life sciences study design

All studies must disclose on these points even when the disclosure is negative.

Sample size	61 participants were included in this study. Participants were divided into separate Discovery (N = 33) and Replication (N = 28) samples. The sample size of the Discovery sample is comparable to similar studies in the field (eg. Miller et al., 2021 J Neurosci). Here, unlike many neuro-anatomical studies, we also include an additional Replication sample to assess the generalizability of our findings to a new group of participants.
Data exclusions	All participants were included in the initial anatomical labeling. For a few participants, we were unable to identify one sulcus in one or both hemispheres (Discovery: n = 5, Replication: n = 3). We excluded these participants for subsequent analyses to ensure that all statistics were balanced for effects of sulcus and hemisphere.
Replication	We conducted all anatomical labeling and morphological analyses separately in the Discovery and Replication samples. There were consistent morphological results in both samples. Within each sample, all morphological-behavioral models were fit with cross-validation which ensures a robust evaluation of a model within a sample. Additionally, we show that a model generated in the Discovery sample generalized to the independent, Replication sample.
Randomization	For the Discovery sample, N = 33 participants were randomly selected from an existing dataset (Wendelken et al., 2011). Following the definition of sulci in this sample, we selected an additional 28 age-matched participants for the Replication sample. No features other than age were considered in the selection of the Replication sample.
Blinding	Blinding was not applicable in this study as participants were not assigned to separate conditions. However, Discovery and Replication samples were kept separate to prevent any data leakage between groups and all data were analyzed through a single pipeline to ensure minimization of influence from the researchers.

Reporting for specific materials, systems and methods

We require information from authors about some types of materials, experimental systems and methods used in many studies. Here, indicate whether each material, system or method listed is relevant to your study. If you are not sure if a list item applies to your research, read the appropriate section before selecting a response.

Materials & experimental systems

n/a	Involved in the study	
☒	☐	Antibodies
☒	☐	Eukaryotic cell lines
☒	☐	Palaeontology and archaeology
☒	☐	Animals and other organisms
☐	☒	Human research participants
☒	☐	Clinical data
☒	☐	Dual use research of concern

Methods

n/a	Involved in the study	
☒	☐	ChIP-seq
☒	☐	Flow cytometry
☐	☒	MRI-based neuroimaging

Human research participants

Policy information about studies involving human research participants

Population characteristics	Cross-sectional data were collected from human participants between the ages of 6 and 18 years old. Participants were separated into Discovery (N=33; males=16, females=17) and Replication (N=28; males=20, females=8) samples*. Age was comparable between the two groups (Discovery: mean = 12.41, Replication: mean = 12.46, p = 0.93). All participants were screened for neurological impairments, psychiatric illness, history of learning disability, and developmental delay.<br>* Note: Male/Female refers to parent reported gender
Recruitment	During initial data collection, participants were recruited from the general community. For this study, participants were randomly sampled from the existing dataset. Sampling was blind to participant demographics.
Ethics oversight	All participants and their parents gave their informed assent and/or consent to participate in the study, which was approved by the Committee for the Protection of Human Subjects at the University of California, Berkeley.

Note that full information on the approval of the study protocol must also be provided in the manuscript.

Magnetic resonance imaging

Experimental design

Design type	N/A
Design specifications	All participants underwent a high resolution T-1 Weighted structural brain imaging scan. Participants also completed two widely used behavioral tasks to measure Reasoning and Processing speed (Figure 1b).
Behavioral performance measures	Reasoning was measured as total raw score from the WISC-IV Matrix Reasoning task. Matrix Reasoning is an untimed subtest of the WISC-IV in which participants are shown colored matrices with one missing quadrant. The participant is asked to “complete” the matrix by selecting the appropriate quadrant from an array of options. Processing speed was computed from raw scores on the Cross-Out task from the Woodcock-Johnson Psychoeducational Battery-Revised. In this task, the participant is presented with a geometric figure on the left followed by 19 similar figures. The subject places a line through each figure that is identical to the figure on the left of the row. Performance is indexed by the number of rows (out of 30 total rows) completed in 3 minutes.

Acquisition

Imaging type(s)	T1-weighted MPRAGE (structural brain scan)
Field strength	3 Tesla
Sequence & imaging parameters	Brain imaging data were collected on a Siemens 3T Trio system at the University of California Berkeley Brain Imaging Center. High-resolution T1-weighted MPRAGE anatomical scans (TR=2300ms, TE=2.98ms, 1×1×1mm voxels) were acquired for cortical morphometric analyses.
Area of acquisition	Whole brain

Diffusion MRI ☐ Used ☒ Not used

Preprocessing

Preprocessing software	Freesurfer 6.0.0 was used for cortical surface reconstruction
Normalization	All sulcal morphological and sulcal-behavioral analyses were performed in native space and no normalization was performed. To generate sulcal probability maps, each sulcal label was transformed from single-subject to fsaverage space via the freesurfer label2label function.
Normalization template	The fsaverage surface (https://surfer.nmr.mgh.harvard.edu/fswiki/FsAverage) was used in the construction of sulcal probability maps.
Noise and artifact removal	All T1-weighted images were visually inspected for scanner artifacts. Each cortical surface reconstruction was visually inspected for segmentation errors, and these were manually corrected when necessary.
Volume censoring	N/A. Volume censoring was not performed in this study.

Statistical modeling & inference

Model type and settings	Repeated measures ANOVAs were used to explore within and between subject variability in sulcal morphology. Multivariate, linear models were used to examine the relationship between sulcal morphology and behavior.
Effect(s) tested	A 2-way repeated measures ANOVA was used to assess differences in sulcal morphology by hemisphere and sulcal type.

Linear models were used to test the association between sulcal morphology and task performance. Linear models were fit with leave-one-out cross validation.

Specify type of analysis: ☐ Whole brain ☒ ROI-based ☐ Both

Anatomical location(s): Regions were defined anatomically according to the most recent and comprehensive sulcal atlas (Petrides, 2019).

Statistic type for inference (See Eklund et al. 2016): For morphological analyses, p-values with a cutoff of $p<0.05$ were used to assess significance of the repeated measures ANOVAs. Generalized eta-squared was calculated to determine effect size. For morphological-behavioral analyses, cross-validated mean-squared error and $R2$ values were used to assess model fits. The Akaike information criterion (AIC) was used for model comparison when appropriate. No functional MRI analyses were performed.

Correction: Correction for multiple comparisons was not required for the above analyses.

Models & analysis

n/a	Involved in the study
☒	☐ Functional and/or effective connectivity
☒	☐ Graph analysis
☐	☒ Multivariate modeling or predictive analysis

Multivariate modeling and predictive analysis: We investigated whether sulcal morphology predicted behavioural task performance. A LASSO regression was used to select variables of interest in the Discovery sample. Cross-validation was used to determine the parameters of the LASSO regression. The features selected in the Discovery sample were then used to construct a linear model to predict task performance in the Replication sample. To evaluate this model, the predictions were compared to alternative nested models. All models were fit using leave-one-out cross validation. Empirical mean-squared error confidence intervals were estimated for each model with a bootstrapping procedure.
